# Dual-hesitant fermatean fuzzy Hamacher aggregation operators and TOPSIS with their application to multi-criteria decision-making

**DOI:** 10.1371/journal.pone.0311580

**Published:** 2024-10-24

**Authors:** Muhammad Amman, Tabasam Rashid, Asif Ali, Olayan Albalawi, Aiedh Mrisi Alharthi

**Affiliations:** 1 Department of Mathematics, School of Sciences, University of Management and Technology, Lahore, Pakistan; 2 Department of Mathematics, Faculty of Science and Technology, Virtual University of Pakistan, Lahore, Pakistan; 3 Department of Statistics, Faculty of Science, University of Tabuk, Tabuk, Saudi Arabia; 4 Department of Mathematics, Turabah University College, Taif University, Taif, Saudi Arabia; Air Force Engineering University, CHINA

## Abstract

The concept of the Dual-hesitant fermatean fuzzy set (DHFFS) represents a significant advancement in practical implementation, combining Fermatean fuzzy sets and Dual-hesitant sets. This new structure uses membership and non-membership hesitancy and is more adaptable for arriving at values in a domain. Since it has the capability to treat multiple fuzzy sets over the degrees of membership and non-membership, the DHFFS greatly improves the flexibility of approaches to tackle multiple-criteria decision-making (MCDM) problems. By applying generalized T‐norm (*T*) and T‐conorm (T*) operation, improved union and intersection formulas are derived. The proposed work adopts Hamacher operations such as Hamacher T-conorm (HT*) and Hamacher T-norm (*HT*) that are more efficient than conventional techniques. New aggregation operators such as Hamacher weighted arithmetic, geometric, power arithmetic, and power geometric are developed for DHFFS. These operators are most beneficial when dealing with a MCDM issue. A case study is used to demonstrate the approachs’ accuracy and effectiveness in real-world decision-making. The comparative and sensitivity analysis results show that these operators are more effective than traditional methods. These results show that the proposed methods are efficient and can be applied in large-scale decision-making processes, strengthening the solutions’ practical implications.

## Introduction

Nowadays, there is uncertainty in various fields, particularly science and technology. Furthermore, uncertainty present in information is far more convoluted against a backdrop of big data. Consequently, Zadeh [1] developed the concept of fuzzy sets (FSs). Due to its distinctive properties, FS has a wide variety of implementations. Although this is inaccurate in practice, FS philosophy defines an object’s degree of non-membership (*ν*) as the counterpart of its degree of membership (*μ*). Later, Atanassov [2] investigated the notion of an intuitionistic fuzzy set (IFS), an enhanced version of fuzzy set theory that can easily explain ambiguous data for multi-criteria decision‐making (MCDM) issues. Since IFSs may be represented by both the degree of *μ* and the degree of *ν*, with their sum being limited to only one, they can more effectively portray the uncertainty of data. FSs come in very handy while dealing with imprecision and indeterminacy as it has the vast capacity to deal with the *μ* and *ν* degree present in an element related to IFS concurrently. To further amplify the IFS idea, some MCDM methods were suggested, such as Liu, Guo, and Garg introducing the notion of some MCDM methods [3–5] based on IFSs, been proposed. Pythagorean fuzzy sets (PFSs), were introduced by Yager to improve the notion of IFSs. This theory has been implemented by multiple researchers in various fields. Yager presented a beneficial method regarding Pythagorean fuzzy aggregation operators [6]. Furthermore, to handle a collaborative-based recommender system, Reformat and Yager [7] utilized the PFNs. Peng described the basic features of PF aggregation operators [8]. After utilizing the information from PFS, Peng, and Yang [9] introduced two very primal arithmetic operations: division and subtraction. Furthermore, they researched their respective traits, including boundness, idempotency, and monotonicity. The practical application of PFS has also been made, such as in the investment decision by Garg [10], the problem of the investment bank of Asia for choosing the best candidate solved by Ren et al. [11] and Wang et al. [12] presented some research on the uncertainty measures for PFS and validate it with MCDM application. Furthermore, Zhang et al. [13] conducted a study on correlation coefficients of PFS and their applications. Similar research within the PFS domain has been explored extensively, as evident from existing literature [14–17]. However, this area has plenty of room for further exploration and investigation. The FS theory is the most common set theory used for MCDM issues. Practically, it takes a lot of work for decision-makers to present their opinions under certain limitations. One limitation is that the FST can only be applicable when the sum of *μ* and *ν* values ≤1. Thus, to avoid complexities and errors caused by such limitations, Senapati and Yager [18] introduced a new set called the Fermatean Fuzzy Set (FFS) that mainly fulfills the condition wherein the cube of a degree of *μ* and *ν* combined is less than or equals one.

While proving the rationality of this idea of FFS, Senapati, and Yager performed an analysis of a numerical example, that is, when one plans to exhibit his inclination of a particular value of a choice *x*_*i*_ within a defined condition, *C*_*j*_. We accurately get .7 + .75 > 1, so following that, the boundary condition of an IFS remains dissatisfied. In addition to this, for the case when (.7)^2^ + (.75)^2^ = .49 + .5625 = 1.0525 > 1, that does not execute the limited circumstances of a PFS. However, it is clear to see (.7)^3^+ (.75)^3^ = .343 + .422 = .7648 < 1, which is a reasonable justification for the suggestion of a new fuzzy set division called FFS. It is also vital here that we mention that the class present under this kind of FSs has a higher capability to apprehend the vagueness than IFSs and PFSs. Furthermore, they are highly capable of managing a higher degree of doubt. The use of MADM is Broad-ranging in multiple fields of sciences, such as the introduction of IF weighted averaging and IF ordered weighted averaging operators by Xu and Xia [19], Kumar and Chen [20]. On these advancements, the emergence of FFS has created more opportunities to deal with even more challenging decision-making problems, as presented in some research papers [21, 22].

The main appeal of fuzzy information aggregation operators is noteworthy in research fields and holds significant consideration in the scientific community. The generalization of algebraic and Einstein T* and *T* [23–25], HT* and *HT* [26, 27] are omnipresent and flexible. Senapati and Yager [28] established four advanced kinds of weighted aggregation operators for FFS such as Fermatean fuzzy weighted average (FFWA), weighted power average (FFWPA), geometric average (FFWGA) and power geometric average (FFWPGA) respectively, to aggregate different fermatean fuzzy numbers (FFNs).

## Literature review

This section reviews the key development within fuzzy set theory has resulted in definitions of different generalizations, each designed to handle different types of uncertainty in decision-making. One such development is the hesitant fuzzy set (HFS), Torra completed the task of generalizing the basic concept of FS notion up to the HFS idea [29]. That latest set held the power to deal with circumstances, the new set of the FS can handle situations such as the complexity information of the degree of *μ* does not deviate from the boundary of error or a specific probability division of presumed values. Although, it does root in the hesitation between multiple values [30]. Thus the HFS can accurately exhibit and transmit people’s hesitation to express their preferences for things, in comparison to the FS and various generalizations [31, 32]. Later, HFS and IFS were conjoined to gain a different HFS known as intuitionistic hesitant fuzzy set (IHFS) and used to solve many MCDM applications [33]. The prime idea is the formation of a situation where researchers hesitate with a set of degrees of *μ* and *ν* rather than an individual degree of *μ* and *ν*, and they need to represent hesitation of this kind. In [34, 35], the concept of a dual-hesitant fuzzy set (DHFS) was further developed and associated with specific attributes.

Attaullah et al. in [36] introduced the research, which has the objective of establishing a methodology using the Fermatean hesitant fuzzy rough set, which combines the Fermatean fuzzy set, hesitant fuzzy set, and rough set to be applied in green supplier selection in the process industry. In this work [37], a new MCDM technique based on the dual hesitant fuzzy assessments as well as the Frank aggregation operators to deal with uncertainty and hesitance in the decision-making process is proposed. Ali et al. [38] enhance the group decision-making by proposing extending the DHFS model by integrating soft expert sets to develop the dual hesitant fuzzy soft expert sets for effectively addressing the uncertainty. It is worth mentioning that Probabilistic hesitant fuzzy sets outperforms the HF approach due to the problem of losing the preference information of all the decision-makers. Due to this advantage, Probabilistic hesitant fuzzy sets is a research that has attracted significant attention in [39]. The original Technique for Order of Preference by Similarity to Ideal Solution (TOPSIS) has been extended to fuzzy TOPSIS to handle imprecision in the assessors’ rating. This fuzzy extension is another technique that has been used extensively in the last decade to address MCDM issues and, in recent years, has attracted the interest of numerous investigators who have proposed different variants and improvements to the method [40]. To overcome the encountered weaknesses in applying TOPSIS for the ranking of alternatives in a Hesitant Fuzzy *β*-Covering Approximation Space, Jin et al. [41] extend the strategy by specifying hesitant fuzzy relations, creating covering rough set models for hesitant fuzzy sets and providing a new method of weight assignments. Likewise, the Multi-criteria optimization and compromise solution (VIKOR) method has been extended for hesitant fuzzy environments, which provided a better option from uncertain environments regarding proximity to the ideal solution [42]. The extension of evaluation based on distance from average solution (EDAS) method has also been used to address hesitant fuzzy information through an alternative ranking that averages the distance between the solutions [43].

Regarding MCDM, dealing with complex and uncertain information is considered critical. Fuzzy set theories of the classical form could be more effective in modeling two types of hesitations and other subtle forms of uncertainties observed in practice. The very nature of fuzzy data amplifies the difficulty levels for processing and data aggregation, and the problems multiply, given the complexity of decision-making. The scenarios expand in numbers and complexity. On the other hand, introducing new methodologies, such as the DHFFSs, to the already popular decision-making models like TOPSIS entails another challenge since it requires appropriate consideration to ensure coherence and credibility. This work fills the gap of the need to establish complex fuzzy models that best mimic the complexities of the decision criteria. Thus, it is essential to use methodologies to make these models more scalable, especially for decision-making purposes, and to apply them to broad areas, including the management of energy resources, among the key ones. The benefits of this research are that it can improve the accuracy of decision-making by using DHFFSs and accompanying aggregation operators, illustrates its functionality by a case of choosing a country for a gas pipeline cooperation, and shows better efficiency in comparison with straightforward techniques as it deals with parallel hesitation and preserves continuous assessment of the options. Our method also addresses the challenge of rank reversal, ensuring that the rankings of alternatives remain stable even when new options are introduced, thereby enhancing decision-making reliability. These contributions make the present work valuable for enhancing the theoretical and practical development of MCDM, serving as tools that reflect the essence of real-world decisions.

The following are the main causes that motivated us to conduct this study.

- As we can see from the study above, most of the Fermatean fuzzy aggregation operators currently in use base their operations on the algebraic product and algebraic sum of FFSs. A general t-norm and a general t-conorm can be used to build a generalized union and an intersection on FFSs.
- Hamacher operations are suitable substitutes for the algebraic product and algebraic sum, including the Hamacher product and Hamacher sum. The study of aggregation operators based on Hamacher operations and their application to multiple attribute group decision-making issues has significant implications.
- The construction of several dual hesitant fermatean fuzzy Hamacher aggregation operators, driven by the standard Hamacher operator and the Fermatean fuzzy set, are more effective and scientific methods of expressing assessment data we suggested for MCDM problems.

### Contributions

The contributions of the proposed research are as follows:

- The first significant finding of this study is the new method of producing DHFFSs by integrating the concept of FFS with DHFS. This new framework assists decision-makers to represent and interpret the hesitant and uncertain decision information correctly, making it appropriate for realistic and complicated decision-making conditions.
- Furthermore, we formulate the operational laws, score and accuracy functions, and distance measures pertaining to Dual-Hesitant Fermatean fuzzy numbers.
- The works for the formulation of Dual-hesitant Fermatean fuzzy weighted aggregation operators (DHFFWAOs) are established with the help of HT and HT* operations. These operators are generally superior at accumulating the sort of vague and hesitant information.
- To solve the MCDM problems with uncertain information, this study proposes a TOPSIS method under the DHFFS environment.
- One essential contribution of this paper is the development of a method resistant to rank reversal, maintaining stable and consistent rankings despite changes in the set of alternatives.
- The developed TOPSIS approach is implemented on a case study of the gas pipeline project of Pakistan with neighboring countries for future gas supply, demonstrating its practicality and managing capacity.

The rest of the paper is structured as follows. In part 2, we discussed already defined concepts. In part 3, we have managed to introduce Dual-hesitant fermatean fuzzy hamacher aggregation operators and analyzed some appealing attributes of the suggested operators and some essential properties of them. Moreover, in part 4, we implemented proposed operators to a case study for decision-making issues whose roots lie in the Hamacher AOs with fermatean fuzzy information. The comparative analysis is discussed in part 5, while sensitivity analysis and rank reversal are in part 6. In Part 7, we explore the potential for scalability of the outlined technique, and in Part 8, we present managerial implications. Limitation of the study and direction for further research is presented in part 9. Last of all, Part 10 sums up the whole paper and presents its main conclusions.

## Preliminaries

In this section, following are some definitions of certain terms used in this article and Table 1 represents the list of variables used in this paper.

**Table 1:** List of variables.

Variables	Description
*☋*	Universal set
A	Attribute set
I	Intuitionistic fuzzy set
P	Pythagorean fuzzy set
F	Fermatean fuzzy set
*μ*, *ν*	Membership and Non-membership
*σ*, *τ*	Fuzzy numbers
*ϕ*	Level of indefiniteness
♇	Dual hesitant fermatean fuzzy set
*S*_⊺_(♇)	Score function of DHFFS
*acc*(♇)	Accuracy function of DHFFS
${\hat{D}}_{H}$	Hamming distance
${\hat{D}}_{E}$	Euclidean distance
*T*	T-norm
T*	T-conorm
*Δ*	Set of alternatives
∁	Set of criteria

**Definition 0.1** *(Atanassov, 2012* [44]*) Let there be a set ☋ with* A *be the set of attributes. IFS* I *on universal set ☋ is shown as* $I=\{u,\sigma_{I}\left(u\right),\tau_{I}\left(u\right)|u\in ☋\}$. *Respectively, the degree of μ and ν defined by functions* $\sigma_{I}\left(u\right):☋\to \left[0,1\right]$ *and* $\tau_{I}\left(u\right):☋\to \left[0,1\right]$ *of the element u* ∈ *☋ to the set* I, *which fulfils the required constraint* $0\leq \sigma_{I}\left(u\right)+\tau_{I}\left(u\right)\leq 1,\forall u\in ☋$. *So, the level of indefiniteness of u* ∈ *☋ is defined as* $\phi_{I(u)}=1-\sigma_{I}\left(u\right)-\tau_{I}\left(u\right).$

**Definition 0.2** *(Yager, 2013* [6]*) Pythagorean Fuzzy Set* P *can be defined, Let univeral set ☋ and PFS as* $P=\{u,\sigma_{P}\left(u\right),\tau_{P}\left(u\right)|u\in ☋\}$. *Consequently as, the degree of μ and ν can be defined by functions* $\sigma_{P}\left(u\right):☋\to \left[0,1\right]$ *and* $\tau_{P}\left(u\right):☋\to \left[0,1\right]$ *of the element u* ∈ *☋ to the set* P, *which satisfies the required constraint* $0\leq {\left(\sigma_{P}\left(u\right)\right)}^{2}+{\left(\tau_{P}\left(u\right)\right)}^{2}\leq 1,\forall u\in ☋$. *So, the level of indefiniteness of u* ∈ *☋ is defined* $\phi_{P(u)}=\sqrt{1-{\left(\sigma_{P}\left(u\right)\right)}^{2}-{\left(\tau_{P}\left(u\right)\right)}^{2}}.$ *To fulfill the condition* ${\left(\sigma_{P}\left(u\right)\right)}^{2}+{\left(\tau_{P}\left(u\right)\right)}^{2}$ *Yager introduced the PFS which is intercepted degree of μ and ν*.

**Definition 0.3** *(Senapati and Yager, 2020* [3]*) A Fermatean fuzzy set* F *in universe of discourse ☋ is an object whose form is shown as* $F=\{u,\sigma_{F}\left(u\right),\tau_{F}\left(u\right)|u\in ☋\}$, *where* $\sigma_{F}\left(u\right):☋\to \left[0,1\right]$ *and* $\tau_{F}\left(u\right):☋\to \left[0,1\right]$, *with the condition* $0\leq {\left(\sigma_{F}\left(u\right)\right)}^{3}+{\left(\tau_{F}\left(u\right)\right)}^{3}\leq 1,$ *for all u* ∈ *☋*. *Respectively, the numbers* $\sigma_{F}\left(u\right)$ *and* $\tau_{F}\left(u\right)$ *denotes the degree of μ and ν of u in set* F. *So, the level of indefiniteness of u* ∈ *☋ is defined as* $\phi_{F(u)}=\sqrt[{3}]{1-{\left(\sigma_{F}\left(u\right)\right)}^{3}-{\left(\tau_{F}\left(u\right)\right)}^{3}}.$

With reference to Table 2 the differentiation between IFS, PFS and FFS is provided. According to Table 2 that shows how FFS is better approach then IFS and PFS.

**Table 2:** IFSs, PFSs and FFSs.

IFSs	PFSs	FFSs
0 ≤ *σ* + *τ* ≤ 1	0 ≤ *σ*^2^ + *τ*^2^ ≤ 1	0 ≤ *σ*^3^ + *τ*^3^ ≤ 1
*σ* + *τ* + *ϕ* = 1	*σ*^2^ + *τ*^2^ + *ϕ*^2^ = 1	*σ*^3^ + *τ*^3^ + *ϕ*^3^ = 1
*ϕ* = 1 − (*σ* + *τ*)	$\phi =\sqrt{1-[\sigma^{2}+\tau^{2}]}$	$\phi =\sqrt[{3}]{1-\sigma^{3}-\tau^{3}}$

**Definition 0.4** *(Senapati and Yager, 2020* [3]*) Score function of any FFS* $F=(\sigma_{F},\tau_{F})$ *is defined as*,

$$\begin{array}{c} S_{⊺}\left(F\right)=\left(\sigma_{F}^{3}-\tau_{F}^{3}\right) \end{array}$$

*where for any FFS function* $S_{⊺}\left(F\right)\in \left[-1,1\right]$.

## Dual-hesitant fermatean fuzzy set (DHFFS)

In the following section of this research paper, we introduce the fundamental concepts of the general form of the FFS [45, 46] and DHFS [34], known as DHFFS. These DHFFSs are constructed using two components: the function of hesitant *μ* and *ν*. This approach offers greater flexibility in assigning values to elements within the domain, requiring handling multiple types of hesitant values.

**Definition 0.5** *Let there be a set A*, *then DHFFS on A can be defined as*:

$$\begin{array}{c} ♇=(<\hbar_{f(a)},{\hat{g}}_{f(a)}>|a\in A) \end{array}$$

*and ℏ*_*f*(*a*)_ *and* ${\hat{g}}_{f(a)}$ *be a couple of sets having discrete values in* [0, 1]. *degree of μ and ν is shown here ℏ*_*f*(*a*)_ *and* ${\hat{g}}_{f(a)}$ *of element a* ∈ *A to the set ♇ respectively. It has condition*

$$\begin{array}{c} 0\leq \sigma^{+3}+\tau^{+3}\leq 1 \end{array}$$

*where σ* ∈ ℏ_*f*(*a*)_ *and* $\tau \in {\hat{g}}_{f(a)}$, ∀*a* ∈ *A*.

$♇\left(a\right)=\left(\hbar_{f(a)},{\hat{g}}_{f(a)}\right)$ *is called dual hesitant fermatean fuzzy number (DHFFN) or ♇* = (*h*, *g*) *with*

$$\begin{array}{c} \sigma \in \hbar ,\tau \in \hat{g},0\leq \sigma ,\tau \leq 1, \end{array}$$

$$\begin{array}{c} 0\leq \sigma^{+3}+\tau^{+3}\leq 1, \end{array}$$

$$\begin{array}{c} \sigma^{+}\in \hbar_{f(a)}^{+}=max\left\{\sigma \right\},\tau^{+}\in {\hat{g}}_{f(a)}^{+}=max\left\{\tau \right\}, \end{array}$$

**Definition 0.6** *Let* $♇=(\hbar ,\hat{g})$ *be a DHFFS then score and accuracy of* $\left(\hbar ,\hat{g}\right)$ *can be found by*

$$\begin{array}{c} S_{⊺}\left(♇\right)=\frac{1}{3}(1+\frac{1}{\#\hbar }\sum_{\sigma \in h}\sigma^{3}-\frac{1}{\#\hat{g}}\sum_{\tau \in h}\tau^{3}) \end{array}$$ (0.1)

$$\begin{array}{c} acc\left(♇\right)=\frac{1}{\#\hbar }\sum_{\sigma \in h}\sigma^{3}+\frac{1}{\#\hat{g}}\sum_{\tau \in h}\tau^{3} \end{array}$$ (0.2)

*where #ℏ and* $\#\hat{g}$ *are the total number of elements in ℏ and* $\hat{g}$ . *Now* ${♇}_{i}=\left(\hbar_{i},{\hat{g}}_{i}\right),\left(i=1,2\right)$ *be any two DHFFNs then*,

*1. If* $S_{⊺}\left({♇}_{1}\right)>S_{⊺}\left({♇}_{2}\right),$ *then ♇*_1_ *is superior to ♇*_2_ *written as ♇*_1_ > *♇*_2_,
*2. If* $S_{⊺}\left({♇}_{1}\right)=S_{⊺}\left({♇}_{2}\right),$ *then*
*(i) If ♇*(*♇*_1_) < *♇*(*♇*_2_), *then ♇*_1_ *is inferioir to ♇*_2_,
*(ii) If ♇*(*♇*_1_) > *♇*(*♇*_2_), *then ♇*_1_ *is superior to ♇*_2_,
*(iii) If ♇*(*♇*_1_) = *♇*(*♇*_2_), *then ♇*_1_ *is equivalent to ♇*_2_, *♇*_1_ ≅ *♇*_2_.

**Definition 0.7** *Suppose there be* $♇,{♇}_{1},{♇}_{2}=\left\{\hbar ,\hat{g}\right\},\left\{\hbar_{1},{\hat{g}}_{1}\right\},\left\{\hbar_{2},{\hat{g}}_{2}\right\}$ *any three DHFFNs respectivley. Few laws are constructed as*:

*1.* ${♇}^{\propto }=\cup_{\sigma \in \hbar ,\tau \in \hat{g}}\{{\left\{\sigma \right\}}^{\propto },\{\sqrt[{3}]{1-{\left(1-\tau^{3}\right)}^{\propto }}\}\},\propto >0$

*2.* $\propto ♇=\cup_{\sigma \in \hbar ,\tau \in \hat{g}}\{\{\sqrt[{3}]{1-{\left(1-\sigma^{3}\right)}^{\propto }}\},{\left\{\tau \right\}}^{\propto }\},\propto >0$

*3.* ${♇}_{1}⊕{♇}_{2}=\cup_{\sigma_{1}\in \hbar_{1},\sigma_{2}\in \hbar_{2},\tau_{1}\in {\hat{g}}_{1},\tau_{2}\in {\hat{g}}_{2}}\{\{\sqrt[{3}]{{\left(\sigma_{1}\right)}^{3}+{\left(\sigma_{2}\right)}^{3}-{\left(\sigma_{1}\right)}^{3}{\left(\sigma_{2}\right)}^{3}}\},\left\{\tau_{1}\tau_{2}\right\}\}$

*4.* ${♇}_{1}⊗{♇}_{2}=\cup_{\sigma_{1}\in \hbar_{1},\sigma_{2}\in \hbar_{2},\tau_{1}\in {\hat{g}}_{1},\tau_{2}\in {\hat{g}}_{2}}\{\{\left\{\sigma_{1}\sigma_{2}\right\},\sqrt[{3}]{{\left(\tau_{1}\right)}^{3}+{\left(\tau_{2}\right)}^{3}-{\left(\tau_{1}\right)}^{3}{\left(\tau_{2}\right)}^{3}}\}\}$

**Definition 0.8** *Let there be two normalized DHFFNs* ${♇}_{1},{♇}_{2}=\left(\hbar_{1},{\hat{g}}_{1}\right),\left(\hbar_{2},{\hat{g}}_{2}\right)$ *respectively, definition of dual-hesitant fermatean fuzzy hamming distance of ♇*_1_ *and ♇*_2_ *is as follows:*

$$\begin{array}{c} {\hat{D}}_{H}\left({♇}_{1},{♇}_{2}\right)=\frac{1}{3}(\frac{1}{\#\hbar }\sum_{\hat{j}=1}^{\#\hbar }|{\left(\sigma_{1}^{+}\right)}^{3}-{\left(\sigma_{2}^{+}\right)}^{3}|+\frac{1}{\#\hat{g}}\sum_{\hat{j}=1}^{\#\hat{g}}|{\left(\tau_{1}^{+}\right)}^{3}-{\left(\tau_{2}^{+}\right)}^{3}\left|+\right|\phi_{1}^{3}-\phi_{2}^{3}|) \end{array}$$ (0.3)

*where ϕ*_1_ *and ϕ*_2_ *be the degree of indeterminancy of ♇*_1_ *and ♇*_2_ *respectively, which can be computed as:*

$$\begin{array}{c} \phi_{1}=\sqrt{1-(\frac{1}{\#\hbar_{1}}\sum_{\sigma \in \hbar_{1}}\sigma^{3}+\frac{1}{\#{\hat{g}}_{1}}\sum_{\tau \in {\hat{g}}_{1}}\tau^{3})}\;,\;\phi_{2}=\sqrt{1-(\frac{1}{\#\hbar_{2}}\sum_{\sigma \in \hbar_{2}}\sigma^{3}+\frac{1}{\#{\hat{g}}_{2}}\sum_{\tau \in {\hat{g}}_{2}}\tau^{3})} \end{array}$$

*Now, dual-hesitant fermatean fuzzy euclidean distance can be computed as:*

$$\begin{array}{c} {\hat{D}}_{E}\left({♇}_{1},{♇}_{2}\right)=\sqrt{\frac{1}{3}(\frac{1}{\#\hbar }\sum_{\hat{j}=1}^{\#\hbar }{\left({\left(\sigma_{1}^{+}\right)}^{3}-{\left(\sigma_{2}^{+}\right)}^{3}\right)}^{2}+\frac{1}{\#\hat{g}}\sum_{\hat{j}=1}^{\#\hat{g}}{\left({\left(\tau_{1}^{+}\right)}^{3}-{\left(\tau_{2}^{+}\right)}^{3}\right)}^{2}+{\left(\phi_{1}^{3}-\phi_{2}^{3}\right)}^{2})} \end{array}$$ (0.4)

*Xu* [47] *proposed the geometric distance model, by applying this model we can generalize the given distance fomrula as:*

$$\begin{array}{c} {\hat{D}}_{G}\left({♇}_{1},{♇}_{2}\right)={\left(\frac{1}{3}(\frac{1}{\#\hbar }\sum_{\hat{j}=1}^{\#\hbar }{\left({\left(\sigma_{1}^{+}\right)}^{3}-{\left(\sigma_{2}^{+}\right)}^{3}\right)}^{\beta }+\frac{1}{\#\hat{g}}\sum_{\hat{j}=1}^{\#\hat{g}}{\left({\left(\tau_{1}^{+}\right)}^{3}-{\left(\tau_{2}^{+}\right)}^{3}\right)}^{\beta }+{\left(\phi_{1}^{3}-\phi_{2}^{3}\right)}^{\beta })\right)}^{\frac{1}{\beta }} \end{array}$$

*here β is constant and β* > 0. *β can be used to generalize the above distance model*.

*i. If β* = 1, *then the distance model is changed to hamming distance [0.3]*.
*i. If β* = 2, *then the distance model is changed to euclidean distance [0.4]*.

### Hamacher operations

Important concepts in FST include *T* and T*, which are used to describe a modified union and intersection of FSs [48]. The definition and criteria of the terms *T* and T* were provided by Roychowdhury in [49]. Deschrijver presented a modified union and a modified intersection of IFSs based on a *T* and T*. Hamacher also suggested a more inclusive *T* and T*s. The Hamacher product (⊗) and sum (⊕) are cases of *T* and T* are included in the Hamacher operation. These are their definitions:

$$\begin{array}{c} T\left(\propto ,\vartheta \right)=\propto ⊕\vartheta =\frac{\propto +\vartheta -\propto \vartheta -(1-\Psi )\propto \vartheta }{1-(1-\Psi )\propto \vartheta },\Psi >0. \end{array}$$

$$\begin{array}{c} T^{*}\left(\propto ,\vartheta \right)=\propto ⊗\vartheta =\frac{\propto \vartheta }{\Psi +(1-\Psi )(\propto +\vartheta -\propto \vartheta )},\Psi >0. \end{array}$$

Now, if *Ψ* = 1, then hamacher *T* and T* will shortened to algebraic *T* and T* respectively,

$$\begin{array}{c} T(\propto ,\vartheta )=\propto ⊕\vartheta =\propto +\vartheta -\propto \vartheta \end{array}$$

$$\begin{array}{c} T^{*}\left(\propto ,\vartheta \right)=\propto ⊗\vartheta =\propto \vartheta \end{array}$$

Now, if *Ψ* = 2, then hamacher *T* and T* will shortened to Einstein *T* and T* respectively,

$$\begin{array}{c} T\left(\propto ,\vartheta \right)=\propto ⊕\vartheta =\frac{\propto +\vartheta }{1+\propto \vartheta } \end{array}$$ (0.5)

$$\begin{array}{c} T^{*}\left(\propto ,\vartheta \right)=\propto ⊗\vartheta =\frac{\propto \vartheta }{1+(1-\propto )(1-\vartheta )}. \end{array}$$ (0.6)

### Hamacher operations of DHFFS

We describe Hamacher operations about DHFFNs by implementing the basic concept of *HT* and HT*. Furthermore, we introduce the Hamacher aggregation operators in conjunction with DHFFNs. Within this context, we explain the following operators: Dual-hesitant fermatean fuzzy Hamacher weighted averaging (DHFFHWA), weighted geometric (DHFFHWG), power weighted averaging (DHFFHPWA), and power weighted geometric (DHFFHPWG) operators.

**Definition 0.9** *Let there be* $♇=\left(\hbar ,\hat{g}\right),{♇}_{1}=\left(\hbar_{1},{\hat{g}}_{1}\right),{♇}_{2}=\left(\hbar_{2},{\hat{g}}_{2}\right)$ *any three DHFFNs. Driven by the arithmetic aggregation operators as in* [50–55] *the* ⊗ *and the* ⊕, *and the generalized intersection and union on two DHFFNs ♇*_1_ *and ♇*_2_, *Ψ > 0 respectively. Some fundamental Hamacher operations on these DHFFNs can be expressed as:*

*1.*
$$\begin{array}{c} {♇}^{\propto }=\;\begin{array}{c} \cup_{\sigma \in \hbar ,\tau \in \hat{g}}(\frac{\sqrt[{3}]{\Psi }\sigma^{\propto }}{\sqrt[{3}]{{\left[1+\left(\Psi -1\right)\left(1-\sigma^{3}\right)\right]}^{\propto }+\left(\Psi -1\right)\sigma^{3\propto }}},\\ \;\;\sqrt[{3}]{\frac{{\left[1+\left(\Psi -1\right)\left(\tau^{3}\right)\right]}^{\propto }-{\left(1-\tau^{3}\right)}^{\propto }}{{\left[1+\left(\Psi -1\right)\left(\tau^{3}\right)\right]}^{\propto }+\left(\Psi -1\right){\left(1-\tau^{3}\right)}^{\propto }}}) \end{array} \end{array}$$
*2.*
$$\begin{array}{c} \propto \left(♇\right)=\;\begin{array}{c} \cup_{\sigma \in \hbar ,\tau \in \hat{g}}(\sqrt[{3}]{\frac{{\left[1+\left(\Psi -1\right)\left(\sigma^{3}\right)\right]}^{\propto }-{\left(1-\sigma^{3}\right)}^{\propto }}{{\left[1+\left(\Psi -1\right)\left(\sigma^{3}\right)\right]}^{\propto }+\left(\Psi -1\right){\left(1-\sigma^{3}\right)}^{\propto }}},\\ \;\;\frac{\sqrt[{3}]{\Psi }\tau^{\propto }}{\sqrt[{3}]{{\left[1+\left(\Psi -1\right)\left(1-\tau^{3}\right)\right]}^{\propto }+\left(\Psi -1\right)\tau^{3\propto }}}) \end{array} \end{array}$$
*3.*
$$\begin{array}{c} {♇}_{1}⊕{♇}_{2}=\;\begin{array}{c} \cup_{\sigma_{1}\in \hbar_{1},\sigma_{2}\in \hbar_{2},\tau_{1}\in {\hat{g}}_{1},\tau_{2}\in {\hat{g}}_{2}}(\sqrt[{3}]{\frac{\sigma_{1}^{3}+\sigma_{2}^{3}-\sigma_{1}^{3}\sigma_{2}^{3}-\left(1-\Psi \right)\sigma_{1}^{3}\sigma_{2}^{3}}{1-\left(1-\Psi \right)\sigma_{1}^{3}\sigma_{2}^{3}}},\\ \;\;\;\;\;\frac{\tau_{1}\tau_{2}}{\sqrt[{3}]{\Psi +\left(1-\Psi \right)\left(\tau_{1}^{3}+\tau_{2}^{3}-\tau_{1}^{3}\tau_{2}^{3}\right)}}) \end{array} \end{array}$$
*4.*
$$\begin{array}{c} {♇}_{1}⊗{♇}_{2}=\;\begin{array}{c} \cup_{\sigma_{1}\in \hbar_{1},\sigma_{2}\in \hbar_{2},\tau_{1}\in {\hat{g}}_{1},\tau_{2}\in {\hat{g}}_{2}}(\frac{\sigma_{1}\sigma_{2}}{\sqrt[{3}]{\Psi +\left(1-\Psi \right)\left(\sigma_{1}^{3}+\sigma_{2}^{3}-\sigma_{1}^{3}\sigma_{2}^{3}\right)}},\\ \;\;\;\;\;\sqrt[{3}]{\frac{\tau_{1}^{3}+\tau_{2}^{3}-\tau_{1}^{3}\tau_{2}^{3}-\left(1-\Psi \right)\tau_{1}^{3}\tau_{2}^{3}}{1-\left(1-\Psi \right)\tau_{1}^{3}\tau_{2}^{3}}}) \end{array} \end{array}$$

### Dual-hesitant fermatean fuzzy Hamacher arithmetic aggregation operators

Let ${♇}_{i}=\left(\hbar_{i},{\hat{g}}_{i}\right)\left(i=1,2,3,\ldots ,n\right)$ be the members of DHFFNs. DHFFHWA operator becomes a mapping as *z*^*n*^ → *z* or can be written as

$$\begin{array}{c} DHFFHWA_{⋓}\left({♇}_{1},{♇}_{2},{♇}_{3},\ldots ,{♇}_{n}\right)={⊕}_{i=1}^{n}\left(⋓{♇}_{n}\right) \end{array}$$

and here $⋓={\left({⋓}_{1},{⋓}_{2},{⋓}_{3},\ldots ,{⋓}_{n}\right)}^{T}$ shows the weight vector of *♇*_*i*_(*i* = 1, 2, 3, ⋯, *n*) which has conditions i.e, ⋓>0 and $\sum_{i=1}^{n}{⋓}_{i}=1$.

The DHFFHWA operator is defined as

$$\begin{array}{c} \;\begin{array}{c} DHFFHWA_{⋓}\left({♇}_{1},{♇}_{2},{♇}_{3},\ldots ,{♇}_{n}\right)={⊕}_{i=1}^{n}\left({⋓}_{i}{♇}_{i}\right)\\ =\cup_{\sigma_{i}\in \hbar_{i},\tau_{i}\in {\hat{g}}_{i}}(\sqrt[{3}]{\frac{\prod_{i=1}^{n}{\left[1+\left(\Psi -1\right)\left(\sigma_{i}^{3}\right)\right]}^{{⋓}_{i}}-\prod_{i=1}^{n}{\left(1-\sigma_{i}^{3}\right)}^{{⋓}_{i}}}{\prod_{i=1}^{n}{\left[1+\left(\Psi -1\right)\left(\sigma_{i}^{3}\right)\right]}^{{⋓}_{i}}+\left(\Psi -1\right)\prod_{i=1}^{n}{\left(1-\sigma_{i}^{3}\right)}^{{⋓}_{i}}}},\\ \frac{\sqrt[{3}]{\Psi }\prod_{i=1}^{n}\tau_{i}^{{⋓}_{i}}}{\sqrt[{3}]{\prod_{i=1}^{n}{\left[1+\left(\Psi -1\right)\left(1-\tau_{i}^{3}\right)\right]}^{{⋓}_{i}}+\left(\Psi -1\right)\prod_{i=1}^{n}\tau_{i}^{3{⋓}_{i}}}}) \end{array} \end{array}$$ (0.7)

Now, few important properties of DHFFHWA are discussed are as follows:

**Theorem 0.10** *(Idempotency property). Let* ${♇}_{i}=\left(\hbar_{i},{\hat{g}}_{i}\right)\left(i=1,2,3,\ldots ,n\right)$ *be the collection of DHFFNs if they all becomes same, i.e*, ${♇}_{i}=\left(\hbar_{i},{\hat{g}}_{i}\right)=z=\left(h,g\right)$, *∀i then we can write it as*,

$$\begin{array}{c} DHFFHWA_{⋓}\left({♇}_{1},{♇}_{2},{♇}_{3},\ldots ,{♇}_{n}\right)=z. \end{array}$$

*Proof:*

*1. Start with the DHFFHWA Operator Formula:*

$$\begin{array}{c} \;\begin{array}{c} DHFFHWA_{⋓}\left({♇}_{1},{♇}_{2},{♇}_{3},\ldots ,{♇}_{n}\right)=\\ (\sqrt[{3}]{\frac{\prod_{i=1}^{n}{\left[1+\left(\Psi -1\right)\left(\sigma_{i}^{3}\right)\right]}^{{⋓}_{i}}-\prod_{i=1}^{n}{\left(1-\sigma_{i}^{3}\right)}^{{⋓}_{i}}}{\prod_{i=1}^{n}{\left[1+\left(\Psi -1\right)\left(\sigma_{i}^{3}\right)\right]}^{{⋓}_{i}}+\left(\Psi -1\right)\prod_{i=1}^{n}{\left(1-\sigma_{i}^{3}\right)}^{{⋓}_{i}}}},\\ \;\;\;\frac{\sqrt[{3}]{\Psi }\prod_{i=1}^{n}\tau_{i}^{{⋓}_{i}}}{\sqrt[{3}]{\prod_{i=1}^{n}{\left[1+\left(\Psi -1\right)\left(1-\tau_{i}^{3}\right)\right]}^{{⋓}_{i}}+\left(\Psi -1\right)\prod_{i=1}^{n}\tau_{i}^{3{⋓}_{i}}}}) \end{array} \end{array}$$ (0.8)

*2. Assume ♇*_*i*_ = (*h*, *g*) *for all i. Here, σ*_*i*_ = *h and τ*_*i*_ = *g*. *Substituting these into the DHFFHWA formula*,

$$\begin{array}{c} \;\begin{array}{c} DHFFHWA_{⋓}\left({♇}_{1},{♇}_{2},{♇}_{3},\ldots ,{♇}_{n}\right)=\\ (\sqrt[{3}]{\frac{\prod_{i=1}^{n}{\left[1+\left(\Psi -1\right)\left(h^{3}\right)\right]}^{{⋓}_{i}}-\prod_{i=1}^{n}{\left(1-h^{3}\right)}^{{⋓}_{i}}}{\prod_{i=1}^{n}{\left[1+\left(\Psi -1\right)\left(h^{3}\right)\right]}^{{⋓}_{i}}+\left(\Psi -1\right)\prod_{i=1}^{n}{\left(1-h^{3}\right)}^{{⋓}_{i}}}},\\ \;\;\;\frac{\sqrt[{3}]{\Psi }\prod_{i=1}^{n}g^{{⋓}_{i}}}{\sqrt[{3}]{\prod_{i=1}^{n}{\left[1+\left(\Psi -1\right)\left(1-g^{3}\right)\right]}^{{⋓}_{i}}+\left(\Psi -1\right)\prod_{i=1}^{n}g^{3{⋓}_{i}}}}) \end{array} \end{array}$$ (0.9)

*3. Since all inputs are identical, the products in the numerator and denominator are the same. Thus, the fraction simplifies to 1 for both components. Therefore*,

$$\begin{array}{c} \sqrt[{3}]{\frac{1-1}{1+0}}=0 \end{array}$$

*and*

$$\begin{array}{c} \frac{\sqrt[{3}]{\Psi }g}{\sqrt[{3}]{\Psi }}=g \end{array}$$

*Thus*,

$$\begin{array}{c} DHFFHWA_{⋓}\left(\left(h,g\right),\left(h,g\right),\ldots ,\left(h,g\right)\right)=\left(h,g\right) \end{array}$$

*Hence, the property is proved*.

**Theorem 0.11** *(Boundedness property). Let* ${♇}_{i}=\left(\hbar_{i},{\hat{g}}_{i}\right)\left(i=1,2,3,\ldots ,n\right)$ *be a family of DHFFNs and we have z*^−^ = min_1≤*i* ≤ *n*_ *♇*_*i*_ *and z*^+^ = max_1≤*i* ≤ *n*_ *♇*_*i*_ *then*,

$$\begin{array}{c} z^{-}\leq DHFFHWA_{⋓}\left({♇}_{1},{♇}_{2},{♇}_{3},\ldots ,{♇}_{n}\right)\leq z^{+}. \end{array}$$

*Proof:*

*1. Start with the DHFFHWA Operator Formula:*

$$\begin{array}{c} \;\begin{array}{c} DHFFHWA_{⋓}\left({♇}_{1},{♇}_{2},{♇}_{3},\ldots ,{♇}_{n}\right)=\\ (\sqrt[{3}]{\frac{\prod_{i=1}^{n}{\left[1+\left(\Psi -1\right)\left(\sigma_{i}^{3}\right)\right]}^{{⋓}_{i}}-\prod_{i=1}^{n}{\left(1-\sigma_{i}^{3}\right)}^{{⋓}_{i}}}{\prod_{i=1}^{n}{\left[1+\left(\Psi -1\right)\left(\sigma_{i}^{3}\right)\right]}^{{⋓}_{i}}+\left(\Psi -1\right)\prod_{i=1}^{n}{\left(1-\sigma_{i}^{3}\right)}^{{⋓}_{i}}}},\\ \;\;\;\frac{\sqrt[{3}]{\Psi }\prod_{i=1}^{n}\tau_{i}^{{⋓}_{i}}}{\sqrt[{3}]{\prod_{i=1}^{n}{\left[1+\left(\Psi -1\right)\left(1-\tau_{i}^{3}\right)\right]}^{{⋓}_{i}}+\left(\Psi -1\right)\prod_{i=1}^{n}\tau_{i}^{3{⋓}_{i}}}}) \end{array} \end{array}$$ (0.10)

*2. For the first component, the maximum and minimum are determined by the extreme values of σ*_*i*_, *and since the product terms in the fraction are bounded by* $\sigma_{i}^{3}$ *values, it holds that:*

$$\begin{array}{c} z^{-}\leq \sqrt[{3}]{\frac{\prod_{i=1}^{n}{\left[1+\left(\Psi -1\right)\left(\sigma_{i}^{3}\right)\right]}^{{⋓}_{i}}-\prod_{i=1}^{n}{\left(1-\sigma_{i}^{3}\right)}^{{⋓}_{i}}}{\prod_{i=1}^{n}{\left[1+\left(\Psi -1\right)\left(\sigma_{i}^{3}\right)\right]}^{{⋓}_{i}}+\left(\Psi -1\right)\prod_{i=1}^{n}{\left(1-\sigma_{i}^{3}\right)}^{{⋓}_{i}}}}\leq z^{+}. \end{array}$$

*Similarly for the second component involving τ*_*i*_,

$$\begin{array}{c} z^{-}\leq \frac{\sqrt[{3}]{\Psi }\prod_{i=1}^{n}\tau_{i}^{{⋓}_{i}}}{\sqrt[{3}]{\prod_{i=1}^{n}{\left[1+\left(\Psi -1\right)\left(1-\tau_{i}^{3}\right)\right]}^{{⋓}_{i}}+\left(\Psi -1\right)\prod_{i=1}^{n}\tau_{i}^{3{⋓}_{i}}}}\leq z^{+}. \end{array}$$

*Since* $\prod_{i=1}^{n}$ *does not exceed the bounds set by z*^−^ *and z*^+^, *the DHFFHWA is bounded*.

*Hence, the property is proved*.

**Theorem 0.12** *(Monotonicity property). Let* ${♇}_{i}=\left(\hbar_{i},{\hat{g}}_{i}\right)\left(i=1,2,3,\ldots ,n\right)$ *and* $z_{i}^{\prime }=\left(h_{i}^{\prime },g_{i}^{\prime }\right)\left(i=1,2,3,\ldots ,n\right)$ *be two families of DHFFNs, such that* ${♇}_{i}\leq z_{i}^{\prime }$ *∀i then*,

$$\begin{array}{c} DHFFHWA_{⋓}\left({♇}_{1},{♇}_{2},{♇}_{3},\ldots ,{♇}_{n}\right)\leq DHFFHWA_{⋓}\left({♇}_{1}^{\prime },{♇}_{2}^{\prime },\ldots ,{♇}_{n}^{\prime }\right). \end{array}$$

*Proof:*

*1. Assume* ${♇}_{i}\leq z_{i}^{\prime }$ :

*This implies* $\sigma_{i}\leq \sigma_{i}^{\prime }$ *and* $\tau_{i}\leq \tau_{i}^{\prime }$ .

*2. If* $\sigma_{i}\leq \sigma_{i}^{\prime }$ , *then substituting into DHFFHWA Formula gives*

$$\begin{array}{c} \;\begin{array}{c} (\sqrt[{3}]{\frac{\prod_{i=1}^{n}{\left[1+\left(\Psi -1\right)\left(\sigma_{i}^{3}\right)\right]}^{{⋓}_{i}}-\prod_{i=1}^{n}{\left(1-\sigma_{i}^{3}\right)}^{{⋓}_{i}}}{\prod_{i=1}^{n}{\left[1+\left(\Psi -1\right)\left(\sigma_{i}^{3}\right)\right]}^{{⋓}_{i}}+\left(\Psi -1\right)\prod_{i=1}^{n}{\left(1-\sigma_{i}^{3}\right)}^{{⋓}_{i}}}}\leq \\ \;\;\;\sqrt[{3}]{\frac{\prod_{i=1}^{n}{\left[1+\left(\Psi -1\right)\left(\sigma_{i}^{\prime 3}\right)\right]}^{{⋓}_{i}}-\prod_{i=1}^{n}{\left(1-\sigma_{i}^{\prime 3}\right)}^{{⋓}_{i}}}{\prod_{i=1}^{n}{\left[1+\left(\Psi -1\right)\left(\sigma_{i}^{\prime 3}\right)\right]}^{{⋓}_{i}}+\left(\Psi -1\right)\prod_{i=1}^{n}{\left(1-\sigma_{i}^{\prime 3}\right)}^{{⋓}_{i}}}}) \end{array} \end{array}$$ (0.11)

*Similarly, if* $\tau_{i}\leq \tau_{i}^{\prime }$ :

$$\begin{array}{c} \;\begin{array}{c} (\frac{\sqrt[{3}]{\Psi }\prod_{i=1}^{n}\tau_{i}^{{⋓}_{i}}}{\sqrt[{3}]{\prod_{i=1}^{n}{\left[1+\left(\Psi -1\right)\left(1-\tau_{i}^{3}\right)\right]}^{{⋓}_{i}}+\left(\Psi -1\right)\prod_{i=1}^{n}\tau_{i}^{3{⋓}_{i}}}}\leq \\ \;\;\;\frac{\sqrt[{3}]{\Psi }\prod_{i=1}^{n}\tau_{i}^{\prime {⋓}_{i}}}{\sqrt[{3}]{\prod_{i=1}^{n}{\left[1+\left(\Psi -1\right)\left(1-\tau_{i}^{\prime 3}\right)\right]}^{{⋓}_{i}}+\left(\Psi -1\right)\prod_{i=1}^{n}\tau_{i}^{\prime 3{⋓}_{i}}}}) \end{array} \end{array}$$ (0.12)

*Therefore*

$$\begin{array}{c} DHFFHWA_{⋓}\left({♇}_{1},{♇}_{2},\ldots ,{♇}_{n}\right)\leq DHFFHWA_{⋓}\left({♇}_{1}^{\prime },{♇}_{2}^{\prime },\ldots ,{♇}_{n}^{\prime }\right) \end{array}$$

*Hence, the property is proved*.

**Theorem 0.13** *(Commutative property). Let* ${♇}_{i}=\left(\hbar_{i},{\hat{g}}_{i}\right)$ *for i* = 1, 2, …, *n*. *Then:*

$$\begin{array}{c} DHFFHWA_{⋓}\left({♇}_{1},{♇}_{2},\ldots ,{♇}_{n}\right)=DHFFHWA_{⋓}\left({♇}_{\sigma (1)},{♇}_{\sigma (2)},\ldots ,{♇}_{\sigma (n)}\right), \end{array}$$

*where σ is any permutation of* {1, 2, …, *n*}.

*Proof:*

*1. The DHFFHWA formula is symmetric with respect to the input permutations because the weights* ⋓_*i*_ *are fixed and only the permutation of the ♇*_*i*_ *values affects the order*.

*2. Consider any permutation σ of the indices*,

$$\begin{array}{c} \;\begin{array}{c} DHFFHWA_{⋓}\left({♇}_{\sigma (1)},{♇}_{\sigma (2)},\ldots ,{♇}_{\sigma (n)}\right)=\\ (\sqrt[{3}]{\frac{\prod_{i=1}^{n}{\left[1+\left(\Psi -1\right)\left(\sigma_{i}^{3}\right)\right]}^{{⋓}_{i}}-\prod_{i=1}^{n}{\left(1-\sigma_{i}^{3}\right)}^{{⋓}_{i}}}{\prod_{i=1}^{n}{\left[1+\left(\Psi -1\right)\left(\sigma_{i}^{3}\right)\right]}^{{⋓}_{i}}+\left(\Psi -1\right)\prod_{i=1}^{n}{\left(1-\sigma_{i}^{3}\right)}^{{⋓}_{i}}}},\\ \;\;\;\frac{\sqrt[{3}]{\Psi }\prod_{i=1}^{n}\tau_{i}^{{⋓}_{i}}}{\sqrt[{3}]{\prod_{i=1}^{n}{\left[1+\left(\Psi -1\right)\left(1-\tau_{i}^{3}\right)\right]}^{{⋓}_{i}}+\left(\Psi -1\right)\prod_{i=1}^{n}\tau_{i}^{3{⋓}_{i}}}}) \end{array} \end{array}$$ (0.13)

*This expression remains unchanged under permutations because the operators’ components are symmetric functions of the inputs*.

*Hence, the property is proved*.

These proofs demonstrate the fundamental properties of the *Ψ* DHFFHWA operator based on its definition and showing that the operator maintains essential properties like idempotency, boundedness, monotonicity, and commutativity.

Now, we elaborate two specific cases based on parameter *Ψ* DHFFHWA which are as follows:

(1) If *Ψ* = 1, then the DHFFHWA will becomes DHFFWA operator:

$$\begin{array}{c} DHFFHWA_{⋓}\left({♇}_{1},{♇}_{2},{♇}_{3},\ldots ,{♇}_{n}\right)={⊕}_{i=1}^{n}\left({⋓}_{i}{♇}_{i}\right)\\ =\cup_{\sigma_{i}\in \hbar_{i},\tau_{i}\in {\hat{g}}_{i}}(\sqrt[{3}]{1-\prod_{i=1}^{n}{\left(1-\sigma_{i}^{3}\right)}^{{⋓}_{i}}},\prod_{i=1}^{n}\tau_{i}^{{⋓}_{i}}) \end{array}$$ (0.14)

(2) If *Ψ* = 2, then the DHFFHWA will becomes Dual-hesitant fermatean fuzzy einstein weighted average (DHFFEWA) operator:

$$\begin{array}{c} \;\begin{array}{c} DHFFHWA_{⋓}\left({♇}_{1},{♇}_{2},{♇}_{3},\ldots ,{♇}_{n}\right)={⊕}_{i=1}^{n}\left({⋓}_{i}{♇}_{i}\right)\\ =\cup_{\sigma_{i}\in \hbar_{i},\tau_{i}\in {\hat{g}}_{i}}(\sqrt[{3}]{\frac{\prod_{i=1}^{n}{\left[1+(\sigma_{i}^{3})\right]}^{{⋓}_{i}}-\prod_{i=1}^{n}{\left(1-\sigma_{i}^{3}\right)}^{{⋓}_{i}}}{\prod_{i=1}^{n}{\left[1+(\sigma_{i}^{3})\right]}^{{⋓}_{i}}+\prod_{i=1}^{n}{\left(1-\sigma_{i}^{3}\right)}^{{⋓}_{i}}}},\\ \;\;\frac{\sqrt[{3}]{2}\prod_{i=1}^{n}\tau_{i}^{{⋓}_{i}}}{\sqrt[{3}]{\prod_{i=1}^{n}{\left[2-\tau_{i}^{3}\right]}^{{⋓}_{i}}+\prod_{i=1}^{n}\tau_{i}^{3{⋓}_{i}}}}) \end{array} \end{array}$$ (0.15)

### Dual-hesitant fermatean fuzzy Hamacher geometric aggregation operators

In this section, a few of the Hamacher geometric aggregation operators by the application of DHFFNs have been discussed along with their properties. These include DHFFHWG operator.

**Definition 0.14** *Let* ${♇}_{i}=\left(\hbar_{i},{\hat{g}}_{i}\right)\left(i=1,2,3,\ldots ,n\right)$ *be the members of DHFFNs. DHFFHWG operator becomes a mapping as z*^*n*^ → *z or can be written as*

$$\begin{array}{c} DHFFHWG_{⋓}\left({♇}_{1},{♇}_{2},{♇}_{3},\ldots ,{♇}_{n}\right)={⊗}_{i=1}^{n}{\left({♇}_{n}\right)}^{{⋓}_{i}} \end{array}$$

and here $⋓={\left({⋓}_{1},{⋓}_{2},{⋓}_{3},\ldots ,{⋓}_{n}\right)}^{T}$ shows the weight vector of *♇*_*i*_(*i* = 1, 2, 3, ⋯, *n*) which has conditions i.e, ⋓>0 and $\sum_{i=1}^{n}{⋓}_{i}=1$.

The DHFFHWG operator is defined as

$$\begin{array}{c} \;\begin{array}{c} DHFFHWG_{⋓}\left({♇}_{1},{♇}_{2},{♇}_{3},\ldots ,{♇}_{n}\right)={⊗}_{i=1}^{n}{\left({♇}_{i}\right)}^{{⋓}_{i}}\\ =\cup_{\tau_{i}\in {\hat{g}}_{i},\sigma_{i}\in \hbar_{i}}(\frac{\sqrt[{3}]{\Psi }\prod_{i=1}^{n}\sigma_{i}^{{⋓}_{i}}}{\sqrt[{3}]{\prod_{i=1}^{n}{\left[1+\left(\Psi -1\right)\left(1-\sigma_{i}^{3}\right)\right]}^{{⋓}_{i}}+\left(\Psi -1\right)\prod_{i=1}^{n}\sigma_{i}^{3{⋓}_{i}}}},\\ \;\;\sqrt[{3}]{\frac{\prod_{i=1}^{n}{\left[1+\left(\Psi -1\right)\left(\tau_{i}^{3}\right)\right]}^{{⋓}_{i}}-\prod_{i=1}^{n}{\left(1-\tau_{i}^{3}\right)}^{{⋓}_{i}}}{\prod_{i=1}^{n}{\left[1+\left(\Psi -1\right)\left(\tau_{i}^{3}\right)\right]}^{{⋓}_{i}}+\left(\Psi -1\right)\prod_{i=1}^{n}{\left(1-\tau_{i}^{3}\right)}^{{⋓}_{i}}}}) \end{array} \end{array}$$ (0.16)

This fact makes it possible to derive properties of the DHFFHWG operator, including idempotency, boundedness, monotonicity, and commutativity, in a way similar to that already outlined for the DHFFHWA operator. The proofs work in parallel with the same reasoning, although the geometric aggregation framework is used instead of the arithmetic one.

Now, we elaborate two specific cases based on parameter *Ψ* DHFFHWG which are as follows:

(1) If *Ψ* = 1, then the DHFFHWG will becomes DHFFWG operator:

$$\begin{array}{c} DHFFHWG_{⋓}\left({♇}_{1},{♇}_{2},{♇}_{3},\ldots ,{♇}_{n}\right)={⊕}_{i=1}^{n}{\left({♇}_{i}\right)}^{{⋓}_{i}}\\ =\cup_{\tau_{i}\in {\hat{g}}_{i},\sigma_{i}\in \hbar_{i}}(\prod_{i=1}^{n}\sigma_{i}^{{⋓}_{i}},\sqrt[{3}]{1-\prod_{i=1}^{n}{\left(1-\tau_{i}^{3}\right)}^{{⋓}_{i}}}) \end{array}$$ (0.17)

(2) If *Ψ* = 2, then the DHFFHWG will becomes Dual-hesitant fermatean fuzzy einstein weighted geometric (DHFFEWG) operator:

$$\begin{array}{c} \;\begin{array}{cc} DHFFHWG_{⋓}\left({♇}_{1},{♇}_{2},{♇}_{3},\ldots ,{♇}_{n}\right) & ={⊗}_{i=1}^{n}{\left({♇}_{i}\right)}^{{⋓}_{i}}\\ & =\cup_{\tau_{i}\in {\hat{g}}_{i},\sigma_{i}\in \hbar_{i}}(\frac{\sqrt[{3}]{2}\prod_{i=1}^{n}\sigma_{i}^{{⋓}_{i}}}{\sqrt[{3}]{\prod_{i=1}^{n}{\left[2-\sigma_{i}^{3}\right]}^{{⋓}_{i}}+\prod_{i=1}^{n}\sigma_{i}^{3{⋓}_{i}}}},\\ & \;\;\sqrt[{3}]{\frac{\prod_{i=1}^{n}{\left[1+(\tau_{i}^{3})\right]}^{{⋓}_{i}}-\prod_{i=1}^{n}{\left[1-\tau_{i}^{3}\right]}^{{⋓}_{i}}}{\prod_{i=1}^{n}{\left[1+(\tau_{i}^{3})\right]}^{{⋓}_{i}}+\prod_{i=1}^{n}{\left[1-\tau_{i}^{3}\right]}^{{⋓}_{i}}}}) \end{array} \end{array}$$ (0.18)

### Dual-hesitant fermatean fuzzy Hamacher power aggregation operators

Introducing Hamacher power AOs by using DHFFNs, called DHFFHPWA operator.

Yager [56] developed the power averaging (*P*_*A*_) operator as a nonlinear weighted average AO, defined as:

$$\begin{array}{c} P_{A}\left(\propto_{1},\propto_{2},\ldots ,\propto_{n}\right)=\frac{\sum_{i=1}^{n}\left(1+T\left(\propto_{i}\right)\right)\propto_{i}}{\sum_{i=1}^{n}\left(1+T\left(\propto_{i}\right)\right)} \end{array}$$ (0.19)

$T\left(\propto_{i}\right)=\sum_{k=1k\neq i}^{n}S_{t}\left(\propto_{i},\propto_{k}\right),$ and *S*_*t*_(∝_*i*_, ∝_*k*_) shows the support for ∝_*i*_ from ∝_*k*_, which fulfils the properties that are as follows:

i. *S*_*t*_(∝_*i*_, ∝_*k*_) ∈ [0, 1],
ii. *S*_*t*_(∝_*i*_, ∝_*k*_) = *S*_*t*_(∝_*k*_, ∝_*i*_),
iii. *S*_*t*_(∝_*i*_, ∝_*k*_) ≥ *S*_*t*_(*u*, *v*), *if* |∝_*i*_ − ∝_*k*_| < |*u* − *v*|.

The similarity index is essentially a measurement of *S*_*t*_. The two values support one another more strongly the more similar they are. Although when the input arguments are exact values, the power AOs are frequently used [57, 58]. This section will look into the *P*_*A*_ operator in dual-hesitant fermatean fuzzy conditions. We define the DHFFHPWA operator utilizing an equation.

**Definition 0.15** *Let* ${♇}_{i}=\left(\hbar_{i},{\hat{g}}_{i}\right)\left(i=1,2,3,\ldots ,n\right)$ *be the members of DHFFNs*. $⋓={\left({⋓}_{1},{⋓}_{2},{⋓}_{3},\ldots ,{⋓}_{n}\right)}^{T}$ *shows the weight vector of ♇*_*i*_(*i* = 1, 2, 3, ⋯, *n*) *and* ⋓>0, $\sum_{i=1}^{n}{⋓}_{i}=1$ *then, DHFFHPWA operator is defined:*

$$\begin{array}{c} DHFFHPWA_{⋓}\left({♇}_{1},{♇}_{2},{♇}_{3},\ldots ,{♇}_{n}\right)\\ =\frac{{⋓}_{1}\left(1+T\left({♇}_{1}\right)\right){♇}_{1}⊕{⋓}_{2}\left(1+T\left({♇}_{2}\right)\right){♇}_{2}⊕,\ldots ,⊕{⋓}_{n}\left(1+T\left({♇}_{n}\right)\right){♇}_{n}}{\sum_{k=1}^{n}{⋓}_{k}\left(1+\left(T*{♇}_{k}\right)\right)} \end{array}$$

*where* $T\left({♇}_{i}\right)=\sum_{k=1k\neq i}^{n}{⋓}_{k}S_{t}\left({♇}_{i},{♇}_{k}\right),$ *and S*_*t*_(*♇*_*i*_, *♇*_*k*_) *shows the support for ♇*_*i*_ *from ♇*_*k*_, *which fulfils the these properties:*

*i. S*_*t*_(*♇*_*i*_, *♇*_*k*_) ∈ [0, 1],
*ii. S*_*t*_(*♇*_*i*_, *♇*_*k*_) = *S*_*t*_(*♇*_*k*_, *♇*_*i*_),
*iii. S*_*t*_(*♇*_*i*_, *♇*_*k*_) ≥ *S*_*t*_(*u*, *v*), *if* |*♇*_*i*_ − *♇*_*k*_| < |*u* − *v*|.

However, if $⋓={\left(\frac{1}{n},\frac{1}{n},\ldots ,\frac{1}{n}\right)}^{T}$, then DHFFHPWA operator shortened to DHFFHPA operator which is as follows:

$$\begin{array}{c} DHFFHPA({♇}_{1},{♇}_{2},{♇}_{3},\ldots ,{♇}_{n})\\ =\frac{\left(1+T\left({♇}_{1}\right)\right){♇}_{1}⊕\left(1+T\left({♇}_{2}\right)\right){♇}_{2}⊕,\ldots ,⊕\left(1+T\left({♇}_{n}\right)\right){♇}_{n}}{\sum_{k=1}^{n}\left(1+\left(T*{♇}_{k}\right)\right)} \end{array}$$

where $T\left({♇}_{i}\right)=\sum_{k=1k\neq i}^{n}S_{t}\left({♇}_{i},{♇}_{k}\right)$.

Now, according to the given operations of DHFFHNs, theorem [0.16] can be solved.

**Theorem 0.16** *Let* ${♇}_{i}=\left(\hbar_{i},{\hat{g}}_{i}\right)\left(i=1,2,3,\ldots ,n\right)$ *be the family of DHFFNs*. $⋓={\left({⋓}_{1},{⋓}_{2},{⋓}_{3},\ldots ,{⋓}_{n}\right)}^{T}$ *shows the weight vector of ♇*_*i*_(*i* = 1, 2, 3, ⋯, *n*) *and* ⋓>0, $\sum_{i=1}^{n}{⋓}_{i}=1,\Psi >1$ *then, aggregated value of DHFFHPWA operator will also be DHFFHNs*.

$$\begin{array}{c} DHFFHPWA_{⋓}\left({♇}_{1},{♇}_{2},{♇}_{3},\ldots ,{♇}_{n}\right)\\ =\frac{{⋓}_{1}\left(1+T\left({♇}_{1}\right)\right){♇}_{1}⊕{⋓}_{2}\left(1+T\left({♇}_{2}\right)\right){♇}_{2}⊕,\ldots ,{⋓}_{n}\left(1+T\left({♇}_{n}\right)\right){♇}_{n}}{\sum_{k=1}^{n}{⋓}_{k}\left(1+\left(T*{♇}_{k}\right)\right)}\\ =\cup_{\sigma_{i}\in \hbar_{i},\tau_{i}\in {\hat{g}}_{i},i=1,2,3,\ldots ,n} \end{array}$$

$$\begin{array}{c} \;\begin{array}{c} \;\;\;\;\;(\sqrt[{3}]{\frac{\prod_{i=1}^{n}{\left[1+\left(\Psi -1\right)\left(\sigma_{i}^{3}\right)\right]}^{\frac{{⋓}_{i}\left(1+T\left({♇}_{i}\right)\right)}{\sum_{i=1}^{n}{⋓}_{i}\left(1+T\left({♇}_{i}\right)\right)}}-\prod_{i=1}^{n}{\left(1-\sigma_{i}^{3}\right)}^{\frac{{⋓}_{i}\left(1+T\left({♇}_{i}\right)\right)}{\sum_{i=1}^{n}{⋓}_{i}\left(1+T\left({♇}_{i}\right)\right)}}}{\prod_{i=1}^{n}{\left[1+\left(\Psi -1\right)\left(\sigma_{i}^{3}\right)\right]}^{\frac{{⋓}_{i}\left(1+T\left({♇}_{i}\right)\right)}{\sum_{i=1}^{n}{⋓}_{i}\left(1+T\left({♇}_{i}\right)\right)}}+\left(\Psi -1\right)\prod_{i=1}^{n}{\left(1-\sigma_{i}^{3}\right)}^{\frac{{⋓}_{i}\left(1+T\left({♇}_{i}\right)\right)}{\sum_{i=1}^{n}{⋓}_{i}\left(1+T\left({♇}_{i}\right)\right)}}}},\\ \;\;\;\;\;\;\frac{\sqrt[{3}]{\Psi }\prod_{i=1}^{n}\tau_{i}^{\frac{{⋓}_{i}\left(1+T\left({♇}_{i}\right)\right)}{\sum_{i=1}^{n}{⋓}_{i}\left(1+T\left({♇}_{i}\right)\right)}}}{\sqrt[{3}]{\prod_{i=1}^{n}{\left[1+\left(\Psi -1\right)\left(1-\tau_{i}^{3}\right)\right]}^{\frac{{⋓}_{i}\left(1+T\left({♇}_{i}\right)\right)}{\sum_{i=1}^{n}{⋓}_{i}\left(1+T\left({♇}_{i}\right)\right)}}+\left(\Psi -1\right)\prod_{i=1}^{n}\tau_{i}^{\frac{3{⋓}_{i}\left(1+T\left({♇}_{i}\right)\right)}{\sum_{i=1}^{n}{⋓}_{i}\left(1+T\left({♇}_{i}\right)\right)}}}}) \end{array} \end{array}$$ (0.20)

*where* $T\left({♇}_{i}\right)=\sum_{k=1k\neq i}^{n}{⋓}_{k}S_{t}\left({♇}_{i},{♇}_{k}\right)$

The following are a few essential properties of the DHFFHPWA operator, which demonstrate its characteristics.

**Theorem 0.17** *(Idempotency property). If* ${♇}_{i}=♇=\left(\hbar ,\hat{g}\right)$ *for all i* = 1, 2, …, *n*, *then:*

$$\begin{array}{c} DHFFHPWA_{⋓}\left(♇,♇,\ldots ,♇\right)=♇. \end{array}$$

*Proof: Given that* ${♇}_{i}=♇=\left(\hbar ,\hat{g}\right)$ *for all i*, *the operator becomes*,

$$\begin{array}{c} DHFFHPWA_{⋓}\left(♇,♇,\ldots ,♇\right)=\frac{{⋓}_{1}\left(1+T\left(♇\right)\right)♇⊕{⋓}_{2}\left(1+T\left(♇\right)\right)♇⊕\ldots ⊕{⋓}_{n}\left(1+T\left(♇\right)\right)♇}{\sum_{k=1}^{n}{⋓}_{k}\left(1+T\left(♇\right)\right)}. \end{array}$$

*The numerator simplifies as*

$$\begin{array}{c} {⊕}_{i=1}^{n}{⋓}_{i}\left(1+T\left(♇\right)\right)♇=\left(1+T\left(♇\right)\right)♇⊕\left(1+T\left(♇\right)\right)♇⊕\ldots ⊕\left(1+T\left(♇\right)\right)♇. \end{array}$$

*Since ♇ is identical for each i*, *this simplifies to*

$$\begin{array}{c} (1+T(♇))♇=♇. \end{array}$$

*Thus, the numerator is just ♇ scaled by* $\sum_{i=1}^{n}{⋓}_{i}$

$$\begin{array}{c} \sum_{i=1}^{n}{⋓}_{i}\left(1+T\left(♇\right)\right)♇=♇. \end{array}$$

*The denominator is also*

$$\begin{array}{c} \sum_{i=1}^{n}{⋓}_{i}\left(1+T\left(♇\right)\right). \end{array}$$

*Thus*,

$$\begin{array}{c} DHFFHPWA_{⋓}\left(♇,♇,\ldots ,♇\right)=\frac{(1+T(♇))♇}{\left(1+T(♇)\right)}=♇. \end{array}$$

This confirms the idempotency property.

**Theorem 0.18** *(Boundedness Property) Given DHFFNs* ${♇}_{i}=\left(\hbar_{i},{\hat{g}}_{i}\right)$ *for i* = 1, 2, …, *n*, *define z*^−^ = min_*i*_*♇*_*i*_ *and z*^+^ = max_*i*_*♇*_*i*_. *Then*:

$$\begin{array}{c} z^{-}\leq DHFFHPWA_{⋓}\left({♇}_{1},{♇}_{2},\ldots ,{♇}_{n}\right)\leq z^{+}. \end{array}$$

*Proof: The DHFFHPWA operator can be expressed as a weighted combination of the DHFFNs*

$$\begin{array}{c} DHFFHPWA_{⋓}\left({♇}_{1},{♇}_{2},\ldots ,{♇}_{n}\right)=\frac{\sum_{i=1}^{n}{⋓}_{i}\left(1+T\left({♇}_{i}\right)\right){♇}_{i}}{\sum_{k=1}^{n}{⋓}_{k}\left(1+T\left({♇}_{k}\right)\right)}. \end{array}$$

*Since the operator is a weighted sum where* ${⋓}_{i}>0$ *and* $\sum_{i=1}^{n}{⋓}_{i}=1$ , *it is essentially a convex combination of the inputs. This implies*,

$$\begin{array}{c} \underset{i}{\text{min}}{♇}_{i}\leq \frac{\sum_{i=1}^{n}{⋓}_{i}\left(1+T\left({♇}_{i}\right)\right){♇}_{i}}{\sum_{k=1}^{n}{⋓}_{k}\left(1+T\left({♇}_{k}\right)\right)}\leq \underset{i}{\text{max}}{♇}_{i}. \end{array}$$

*Thus, we have*,

$$\begin{array}{c} z^{-}\leq DHFFHPWA_{⋓}\left({♇}_{1},{♇}_{2},\ldots ,{♇}_{n}\right)\leq z^{+}. \end{array}$$

**Theorem 0.19** *(Monotonicity Property) Let* ${♇}_{i}=\left(\hbar_{i},{\hat{g}}_{i}\right)$ *and* ${♇}_{i}^{\prime }=\left(\hbar_{i}^{\prime },{\hat{g}}_{i}^{\prime }\right)$ *for i* = 1, 2, …, *n*, *such that* ${♇}_{i}\leq {♇}_{i}^{\prime }$ *for all i*. *Then*:

$$\begin{array}{c} DHFFHPWA_{⋓}\left({♇}_{1},{♇}_{2},\ldots ,{♇}_{n}\right)\leq DHFFHPWA_{⋓}\left({♇}_{1}^{\prime },{♇}_{2}^{\prime },\ldots ,{♇}_{n}^{\prime }\right). \end{array}$$

*Proof: Since* ${♇}_{i}\leq {♇}_{i}^{\prime }$ *for all i, it follows that*

$$\begin{array}{c} {⋓}_{i}\left(1+T\left({♇}_{i}\right)\right){♇}_{i}\leq {⋓}_{i}\left(1+T\left({♇}_{i}^{\prime }\right)\right){♇}_{i}^{\prime }. \end{array}$$

*Summing these weighted terms for all i, we get*

$$\begin{array}{c} \sum_{i=1}^{n}{⋓}_{i}\left(1+T\left({♇}_{i}\right)\right){♇}_{i}\leq \sum_{i=1}^{n}{⋓}_{i}\left(1+T\left({♇}_{i}^{\prime }\right)\right){♇}_{i}^{\prime }. \end{array}$$

*Since the denominator* $\sum_{k=1}^{n}{⋓}_{k}\left(1+T\left({♇}_{k}\right)\right)$ *is the same in both cases, it follows that*

$$\begin{array}{c} DHFFHPWA_{⋓}\left({♇}_{1},{♇}_{2},\ldots ,{♇}_{n}\right)\leq DHFFHPWA_{⋓}\left({♇}_{1}^{\prime },{♇}_{2}^{\prime },\ldots ,{♇}_{n}^{\prime }\right). \end{array}$$

This confirms the monotonicity property.

**Theorem 0.20** *(Commutative Property)*

*For any permutation π of* {1, 2, …, *n*}:

$$\begin{array}{c} DHFFHPWA_{⋓}\left({♇}_{1},{♇}_{2},\ldots ,{♇}_{n}\right)=DHFFHPWA_{⋓}\left({♇}_{\pi (1)},{♇}_{\pi (2)},\ldots ,{♇}_{\pi (n)}\right). \end{array}$$

*Proof: The DHFFHPWA operator is defined as*,

$$\begin{array}{c} DHFFHPWA_{⋓}\left({♇}_{1},{♇}_{2},\ldots ,{♇}_{n}\right)=\frac{\sum_{i=1}^{n}{⋓}_{i}\left(1+T\left({♇}_{i}\right)\right){♇}_{i}}{\sum_{k=1}^{n}{⋓}_{k}\left(1+T\left({♇}_{k}\right)\right)}. \end{array}$$

*This operator depends only on the values of the ♇*_*i*_ *and not on their order. The weights ⋓*_*i*_ *and the values ♇*_*i*_ *remain the same under any permutation, so the weighted sum remains unchanged*

$$\begin{array}{c} \sum_{i=1}^{n}{⋓}_{i}\left(1+T\left({♇}_{i}\right)\right){♇}_{i}=\sum_{i=1}^{n}{⋓}_{\pi (i)}\left(1+T\left({♇}_{\pi (i)}\right)\right){♇}_{\pi (i)}. \end{array}$$

*Thus*,

$$\begin{array}{c} DHFFHPWA_{⋓}\left({♇}_{1},{♇}_{2},\ldots ,{♇}_{n}\right)=DHFFHPWA_{⋓}\left({♇}_{\pi (1)},{♇}_{\pi (2)},\ldots ,{♇}_{\pi (n)}\right). \end{array}$$

This confirms the commutative property.

Now, two specific cases based on parameter *Ψ* of DHFFHPWA operator are discussed.

(1) If *Ψ* = 1, then the DHFFHPWA will becomes DHFFPWA operator:

$$\begin{array}{c} =\cup_{\sigma_{i}\in \hbar_{i},\tau_{i}\in {\hat{g}}_{i}}(\sqrt[{3}]{1-\prod_{i=1}^{n}{\left(1-\sigma_{i}^{3}\right)}^{\frac{{⋓}_{i}\left(1+T\left({♇}_{i}\right)\right)}{\sum_{i=1}^{n}{⋓}_{i}\left(1+T\left({♇}_{i}\right)\right)}}},\prod_{i=1}^{n}\tau_{i}^{\frac{{⋓}_{i}\left(1+T\left({♇}_{i}\right)\right)}{\sum_{i=1}^{n}{⋓}_{i}\left(1+T\left({♇}_{i}\right)\right)}}) \end{array}$$ (0.21)

(2) If *Ψ* = 2, then the DHFFHPWA will becomes DHFFEPWA operator:

$$\begin{array}{c} \;\begin{array}{c} \;\;\;\;=\cup_{\sigma_{i}\in \hbar_{i},\tau_{i}\in {\hat{g}}_{i}}(\sqrt[{3}]{\frac{\prod_{i=1}^{n}{\left[1+(\sigma_{i}^{3})\right]}^{\frac{{⋓}_{i}\left(1+T\left({♇}_{i}\right)\right)}{\sum_{i=1}^{n}{⋓}_{i}\left(1+T\left({♇}_{i}\right)\right)}}-\prod_{i=1}^{n}{\left(1-\sigma_{i}^{3}\right)}^{\frac{{⋓}_{i}\left(1+T\left({♇}_{i}\right)\right)}{\sum_{i=1}^{n}{⋓}_{i}\left(1+T\left({♇}_{i}\right)\right)}}}{\prod_{i=1}^{n}{\left[1+(\sigma_{i}^{3})\right]}^{\frac{{⋓}_{i}\left(1+T\left({♇}_{i}\right)\right)}{\sum_{i=1}^{n}{⋓}_{i}\left(1+T\left({♇}_{i}\right)\right)}}+\prod_{i=1}^{n}{\left(1-\sigma_{i}^{3}\right)}^{\frac{{⋓}_{i}\left(1+T\left({♇}_{i}\right)\right)}{\sum_{i=1}^{n}{⋓}_{i}\left(1+T\left({♇}_{i}\right)\right)}}}},\\ \;\;\;\;\;\;\;\;\frac{\sqrt[{3}]{2}\prod_{i=1}^{n}\tau_{i}^{\frac{{⋓}_{i}\left(1+T\left({♇}_{i}\right)\right)}{\sum_{i=1}^{n}{⋓}_{i}\left(1+T\left({♇}_{i}\right)\right)}}}{\sqrt[{3}]{\prod_{i=1}^{n}{\left[2-\tau_{i}^{3}\right]}^{\frac{{⋓}_{i}\left(1+T\left({♇}_{i}\right)\right)}{\sum_{i=1}^{n}{⋓}_{i}\left(1+T\left({♇}_{i}\right)\right)}}+\prod_{i=1}^{n}\tau_{i}^{\frac{3{⋓}_{i}\left(1+T\left({♇}_{i}\right)\right)}{\sum_{i=1}^{n}{⋓}_{i}\left(1+T\left({♇}_{i}\right)\right)}}}}) \end{array} \end{array}$$ (0.22)

### Dual-hesitant fermatean fuzzy Hamacher power geometric operators

Introducing Hamacher power geometric operator [59, 60] by using DHFFNs, called DHFFHPWG operator.

**Definition 0.21** *Let* ${♇}_{i}=\left(\hbar_{i},{\hat{g}}_{i}\right)\left(i=1,2,3,\ldots ,n\right)$ *be the members of DHFFNs*. $⋓={\left({⋓}_{1},{⋓}_{2},{⋓}_{3},\ldots ,{⋓}_{n}\right)}^{T}$ *shows the weight vector of ♇*_*i*_(*i* = 1, 2, 3, ⋯, *n*) *and* ⋓>0, $\sum_{i=1}^{n}{⋓}_{i}=1$ *then, DHFFHPWG is given as:*

$$\begin{array}{c} DHFFHPWG_{⋓}\left({♇}_{1},{♇}_{2},{♇}_{3},\ldots ,{♇}_{n}\right)\\ ={♇}_{1}^{\frac{{⋓}_{1}\left(1+T\left({♇}_{1}\right)\right)}{\sum_{i=1}^{n}{⋓}_{1}\left(1+T\left({♇}_{1}\right)\right)}}⊗{♇}_{2}^{\frac{{⋓}_{2}\left(1+T\left({♇}_{2}\right)\right)}{\sum_{i=1}^{n}{⋓}_{2}\left(1+T\left({♇}_{2}\right)\right)}}⊗,\ldots ,⊗{♇}_{n}^{\frac{{⋓}_{n}\left(1+T\left({♇}_{n}\right)\right)}{\sum_{i=1}^{n}{⋓}_{n}\left(1+T\left({♇}_{n}\right)\right)}} \end{array}$$

*where* $T\left({♇}_{i}\right)=\sum_{k=1k\neq i}^{n}{⋓}_{k}S_{t}\left({♇}_{i},{♇}_{k}\right),$ *and S*_*t*_(*♇*_*i*_, *♇*_*k*_) *shows the suppport for ♇*_*i*_ *from ♇*_*k*_, *which fulfils the following three properties:*

*i. S*_*t*_(*♇*_*i*_, *♇*_*k*_) ∈ [0, 1],
*ii. S*_*t*_(*♇*_*i*_, *♇*_*k*_) = *S*_*t*_(*♇*_*k*_, *♇*_*i*_),
*iii. S*_*t*_(*♇*_*i*_, *♇*_*k*_) ≥ *S*_*t*_(*u*, *v*), *if |♇*_*i*_ − *♇*_*k*_| < |*u* − *v*|.

However, if $⋓={\left(\frac{1}{n},\frac{1}{n},\ldots ,\frac{1}{n}\right)}^{T}$, then DHFFHPWG operator shortened to DHFFHPG operator which is as follows:

$$\begin{array}{c} DHFFHPW{\hat{g}}_{⋓}\left({♇}_{1},{♇}_{2},{♇}_{3},\ldots ,{♇}_{n}\right)\\ ={♇}_{1}^{\frac{\left(1+T\left({♇}_{1}\right)\right)}{\sum_{i=1}^{n}\left(1+T\left({♇}_{1}\right)\right)}}⊗{♇}_{2}^{\frac{\left(1+T\left({♇}_{2}\right)\right)}{\sum_{i=1}^{n}\left(1+T\left({♇}_{2}\right)\right)}}⊗,\ldots ,⊗{♇}_{n}^{\frac{\left(1+T\left({♇}_{n}\right)\right)}{\sum_{i=1}^{n}\left(1+T\left({♇}_{n}\right)\right)}} \end{array}$$

where $T\left({♇}_{i}\right)=\sum_{k=1k\neq i}^{n}S_{t}\left({♇}_{i},{♇}_{k}\right)$.

Now, according to the given operations of DHFFHNs, theorem [0.22] can be solved.

**Theorem 0.22** *Let* ${♇}_{i}=\left(\hbar_{i},{\hat{g}}_{i}\right)\left(i=1,2,3,\ldots ,n\right)$ *be the family of DHFFNs*. $⋓={\left({⋓}_{1},{⋓}_{2},{⋓}_{3},\ldots ,{⋓}_{n}\right)}^{T}$ *shows the weight vector of ♇*_*i*_(*i* = 1, 2, 3, ⋯, *n*) *and* ⋓>0, $\sum_{i=1}^{n}{⋓}_{i}=1,\Psi >1$ *then, aggregated value of DHFFHPWG operator will also be DHFFHNs*.

$$\begin{array}{c} DHFFHPWG_{⋓}\left({♇}_{1},{♇}_{2},\ldots ,{♇}_{n}\right)\\ ={♇}_{1}^{\frac{{⋓}_{1}\left(1+T\left({♇}_{1}\right)\right)}{\sum_{i=1}^{n}{⋓}_{1}\left(1+T\left({♇}_{1}\right)\right)}}⊗{♇}_{2}^{\frac{{⋓}_{2}\left(1+T\left({♇}_{2}\right)\right)}{\sum_{i=1}^{n}{⋓}_{2}\left(1+T\left({♇}_{2}\right)\right)}}⊗,\ldots ,⊗{♇}_{n}^{\frac{{⋓}_{n}\left(1+T\left({♇}_{n}\right)\right)}{\sum_{i=1}^{n}{⋓}_{n}\left(1+T\left({♇}_{n}\right)\right)}} \end{array}$$

$$\begin{array}{c} \;\begin{array}{c} =\cup_{\sigma_{i}\in \hbar_{i},\tau_{i}\in {\hat{g}}_{i},i=1,2,3,\ldots ,n}(\frac{\sqrt[{3}]{\Psi }\prod_{i=1}^{n}\sigma_{i}^{\frac{{⋓}_{i}\left(1+T\left({♇}_{i}\right)\right)}{\sum_{i=1}^{n}{⋓}_{i}\left(1+T\left({♇}_{i}\right)\right)}}}{\sqrt[{3}]{\prod_{i=1}^{n}{\left[1+\left(\Psi -1\right)\left(1-\sigma_{i}^{3}\right)\right]}^{\frac{{⋓}_{i}\left(1+T\left({♇}_{i}\right)\right)}{\sum_{i=1}^{n}{⋓}_{i}\left(1+T\left({♇}_{i}\right)\right)}}+\left(\Psi -1\right)\prod_{i=1}^{n}\sigma_{i}^{\frac{3{⋓}_{i}\left(1+T\left({♇}_{i}\right)\right)}{\sum_{i=1}^{n}{⋓}_{i}\left(1+T\left({♇}_{i}\right)\right)}}}},\\ \;\;\;\;\;\sqrt[{3}]{\frac{\prod_{i=1}^{n}{\left[1+\left(\Psi -1\right)\left(\tau_{i}^{3}\right)\right]}^{\frac{{⋓}_{i}\left(1+T\left({♇}_{i}\right)\right)}{\sum_{i=1}^{n}{⋓}_{i}\left(1+T\left({♇}_{i}\right)\right)}}-\prod_{i=1}^{n}{\left(1-\tau_{i}^{3}\right)}^{\frac{{⋓}_{i}\left(1+T\left({♇}_{i}\right)\right)}{\sum_{i=1}^{n}{⋓}_{i}\left(1+T\left({♇}_{i}\right)\right)}}}{\prod_{i=1}^{n}{\left[1+\left(\Psi -1\right)\left(\tau_{i}^{3}\right)\right]}^{\frac{{⋓}_{i}\left(1+T\left({♇}_{i}\right)\right)}{\sum_{i=1}^{n}{⋓}_{i}\left(1+T\left({♇}_{i}\right)\right)}}+\left(\Psi -1\right)\prod_{i=1}^{n}{\left(1-\tau_{i}^{3}\right)}^{\frac{{⋓}_{i}\left(1+T\left({♇}_{i}\right)\right)}{\sum_{i=1}^{n}{⋓}_{i}\left(1+T\left({♇}_{i}\right)\right)}}}}.) \end{array} \end{array}$$ (0.23)

*where*, $T\left({♇}_{i}\right)=\sum_{k=1k\neq i}^{n}{⋓}_{k}S_{t}\left({♇}_{i},{♇}_{k}\right)$

As for the properties of the DHFFHPWG operator, they also can be deduced in similar way as that of the DHFFHPWA operator, but within the framework of the geometric aggregation.

Now, two specific cases based on parameter *Ψ* of DHFFHPWG operator are discussed.

(1) If *Ψ* = 1, then the DHFFHPWG will becomes DHFFPWG operator:

$$\begin{array}{c} =\cup_{\sigma_{i}\in \hbar_{i},\tau_{i}\in {\hat{g}}_{i}}(\prod_{i=1}^{n}\sigma_{i}^{\frac{{⋓}_{i}\left(1+T\left({♇}_{i}\right)\right)}{\sum_{i=1}^{n}{⋓}_{i}\left(1+T\left({♇}_{i}\right)\right)}},\sqrt[{3}]{1-\prod_{i=1}^{n}{\left(1-\tau_{i}^{3}\right)}^{\frac{{⋓}_{i}\left(1+T\left({♇}_{i}\right)\right)}{\sum_{i=1}^{n}{⋓}_{i}\left(1+T\left({♇}_{i}\right)\right)}}}) \end{array}$$ (0.24)

(2) If *Ψ* = 2, then the DHFFHPWG will becomes DHFFEPWG operator:

$$\begin{array}{c} \;\begin{array}{c} =\cup_{\sigma_{i}\in \hbar_{i},\tau_{i}\in {\hat{g}}_{i}}(\frac{\sqrt[{3}]{2}\prod_{i=1}^{n}\sigma_{i}^{\frac{{⋓}_{i}\left(1+T\left({♇}_{i}\right)\right)}{\sum_{i=1}^{n}{⋓}_{i}\left(1+T\left({♇}_{i}\right)\right)}}}{\sqrt[{3}]{\prod_{i=1}^{n}{\left[2-\sigma_{i}^{3}\right]}^{\frac{{⋓}_{i}\left(1+T\left({♇}_{i}\right)\right)}{\sum_{i=1}^{n}{⋓}_{i}\left(1+T\left({♇}_{i}\right)\right)}}+\prod_{i=1}^{n}\sigma_{i}^{\frac{3{⋓}_{i}\left(1+T\left({♇}_{i}\right)\right)}{\sum_{i=1}^{n}{⋓}_{i}\left(1+T\left({♇}_{i}\right)\right)}}}},\\ \;\;\sqrt[{3}]{\frac{\prod_{i=1}^{n}{\left[1+(\tau_{i}^{3})\right]}^{\frac{{⋓}_{i}\left(1+T\left({♇}_{i}\right)\right)}{\sum_{i=1}^{n}{⋓}_{i}\left(1+T\left({♇}_{i}\right)\right)}}-\prod_{i=1}^{n}{\left(1-\tau_{i}^{3}\right)}^{\frac{{⋓}_{i}\left(1+T\left({♇}_{i}\right)\right)}{\sum_{i=1}^{n}{⋓}_{i}\left(1+T\left({♇}_{i}\right)\right)}}}{\prod_{i=1}^{n}{\left[1+(\tau_{i}^{3})\right]}^{\frac{{⋓}_{i}\left(1+T\left({♇}_{i}\right)\right)}{\sum_{i=1}^{n}{⋓}_{i}\left(1+T\left({♇}_{i}\right)\right)}}+\prod_{i=1}^{n}{\left(1-\tau_{i}^{3}\right)}^{\frac{{⋓}_{i}\left(1+T\left({♇}_{i}\right)\right)}{\sum_{i=1}^{n}{⋓}_{i}\left(1+T\left({♇}_{i}\right)\right)}}}}) \end{array} \end{array}$$ (0.25)

### Model of hamacher operators for MCDM with DHFFS

**Fig 1:**
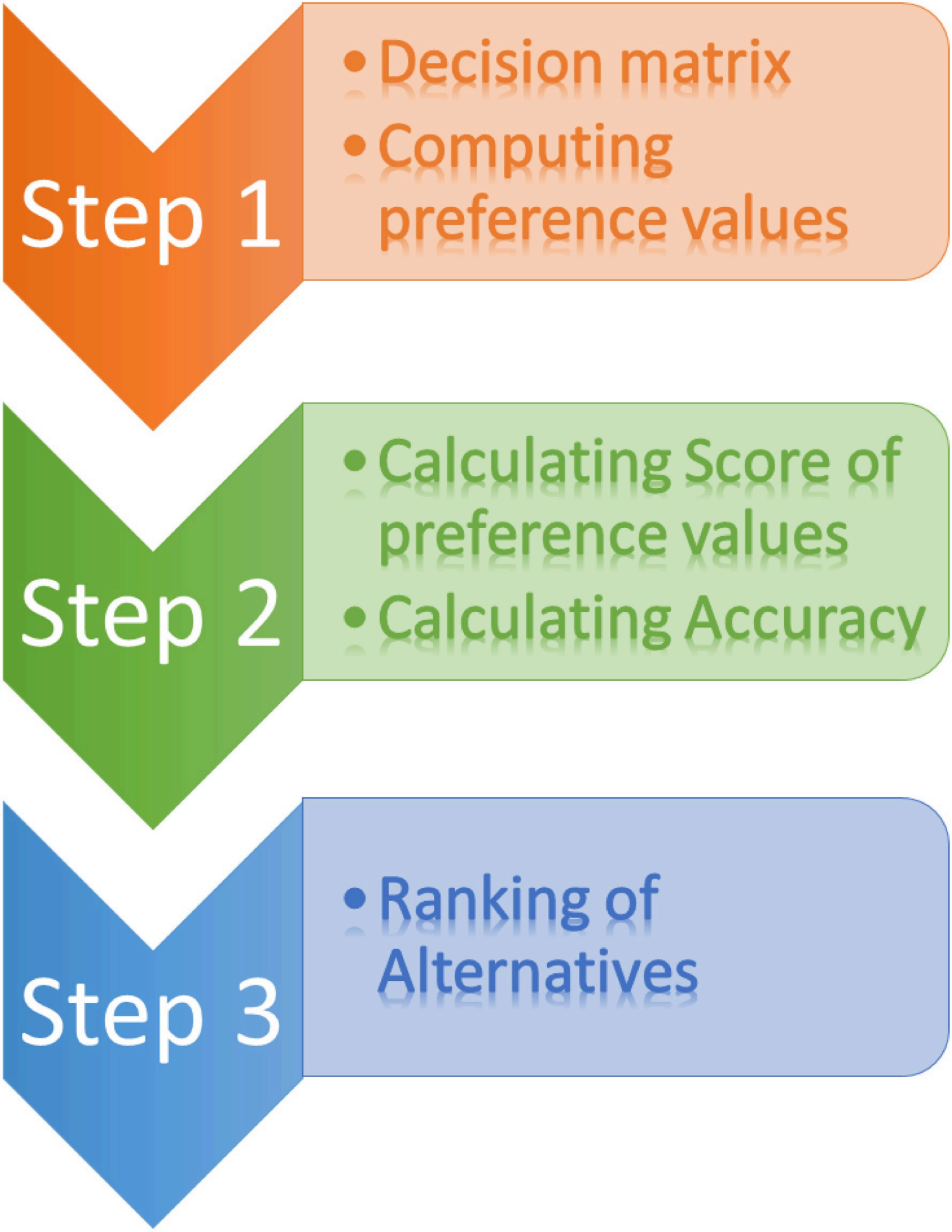
Flowchart of hamacher operators for MCDM with DHFFS.

In this part of the paper, the utilization of DHFFHAOs to sort out the MCDM problems resulting from DHFFNs. For representing the MCDM problems with DHFFNs, the following assumptions are made. Let *Δ*_*i*_ = {*Δ*_1_, *Δ*_2_, ⋯, *Δ*_*m*_} be a discrete set of alternatives, and ∁_*i*_ = {∁_1_, ∁_2_, ⋯, ∁_*n*_} be the state of criteria. Assume that the decision matrix $♇={\left({♇}_{i\hat{j}}\right)}_{m\times n}={\left(\hbar_{i\hat{j}},{\hat{g}}_{i\hat{j}}\right)}_{m\times n}$ is the Dual-hesitant fermatean fuzzy decision matrix, where ${\left({♇}_{i\hat{j}}\right)}_{m\times n}={\left(\hbar_{i\hat{j}},{\hat{g}}_{i\hat{j}}\right)}_{m\times n}\left(i=1,2,3,\ldots ,m,j=1,2,\ldots ,n\right)$ are in the form of DHFFNs. In the following, we apply the DHFFHWA and DHFFHWG operators to the MADM problems with DHFFNs. Fig 1 shows the flowchart of the above-described model.

Step 1. Comprehensive weightages of all the alternatives *Δ* can be derived by utilizing the information obtained from decision matrix *♇* with DHFFHWA and DHFFHWG operators,
  $$\begin{array}{c} {♇}_{i}=DHFFHWA_{{⋓}_{j}}\left({♇}_{i1},{♇}_{i1},\ldots ,{♇}_{in}\right)={⊕}_{\hat{j}=1}^{n}\left({⋓}_{j}{♇}_{i\hat{j}}\right)\\ =\cup_{\sigma_{i}\in \hbar_{i},\tau_{i}\in {\hat{g}}_{i}}(\sqrt[{3}]{\frac{\prod_{i=1}^{n}{\left[1+\left(\Psi -1\right)\left(\sigma_{i}^{3}\right)\right]}^{{⋓}_{i}}-\prod_{i=1}^{n}{\left(1-\sigma_{i}^{3}\right)}^{{⋓}_{i}}}{\prod_{i=1}^{n}{\left[1+\left(\Psi -1\right)\left(\sigma_{i}^{3}\right)\right]}^{{⋓}_{i}}+\left(\Psi -1\right)\prod_{i=1}^{n}{\left(1-\sigma_{i}^{3}\right)}^{{⋓}_{i}}}},\\ \;\;\frac{\sqrt[{3}]{\Psi }\prod_{i=1}^{n}\tau_{i}^{{⋓}_{i}}}{\sqrt[{3}]{\prod_{i=1}^{n}{\left[1+\left(\Psi -1\right)\left(1-\tau_{i}^{3}\right)\right]}^{{⋓}_{i}}+\left(\Psi -1\right)\prod_{i=1}^{n}\tau_{i}^{3{⋓}_{i}}}}) \end{array}$$
  $$\begin{array}{c} {♇}_{i}=DHFFHWG_{{⋓}_{j}}\left({♇}_{i1},{♇}_{i1},\ldots ,{♇}_{in}\right)={⊗}_{\hat{j}=1}^{n}{\left({♇}_{i\hat{j}}\right)}^{{⋓}_{j}}\\ =\cup_{\tau_{i}\in {\hat{g}}_{i},\sigma_{i}\in \hbar_{i}}(\frac{\sqrt[{3}]{\Psi }\prod_{i=1}^{n}\sigma_{i}^{{⋓}_{i}}}{\sqrt[{3}]{\prod_{i=1}^{n}{\left[1+\left(\Psi -1\right)\left(1-\sigma_{i}^{3}\right)\right]}^{{⋓}_{i}}+\left(\Psi -1\right)\prod_{i=1}^{n}\sigma_{i}^{3{⋓}_{i}}}},\\ \;\;\sqrt[{3}]{\frac{\prod_{i=1}^{n}{\left[1+\left(\Psi -1\right)\left(\tau_{i}^{3}\right)\right]}^{{⋓}_{i}}-\prod_{i=1}^{n}{\left(1-\tau_{i}^{3}\right)}^{{⋓}_{i}}}{\prod_{i=1}^{n}{\left[1+\left(\Psi -1\right)\left(\tau_{i}^{3}\right)\right]}^{{⋓}_{i}}+\left(\Psi -1\right)\prod_{i=1}^{n}{\left(1-\tau_{i}^{3}\right)}^{{⋓}_{i}}}}) \end{array}$$
Step 2. Computing the $S_{⊺}\left({♇}_{i}\right)$ and *acc*(*♇*_*i*_), (*i* = 1, 2, 3, ⋯, *m*) of the comprehensive weightage of all the alternatives *Δ*_*i*_ = {*Δ*_1_, *Δ*_2_, *Δ*_3_, ⋯, *Δ*_*m*_}.
Step 3. Ranking of alternatives to obtain best alternative relative to $S_{⊺}\left({♇}_{i}\right)$ and *acc*(*♇*_*i*_) is done in this step.

### TOPSIS for MCDM with DHFFS

**Fig 2:**
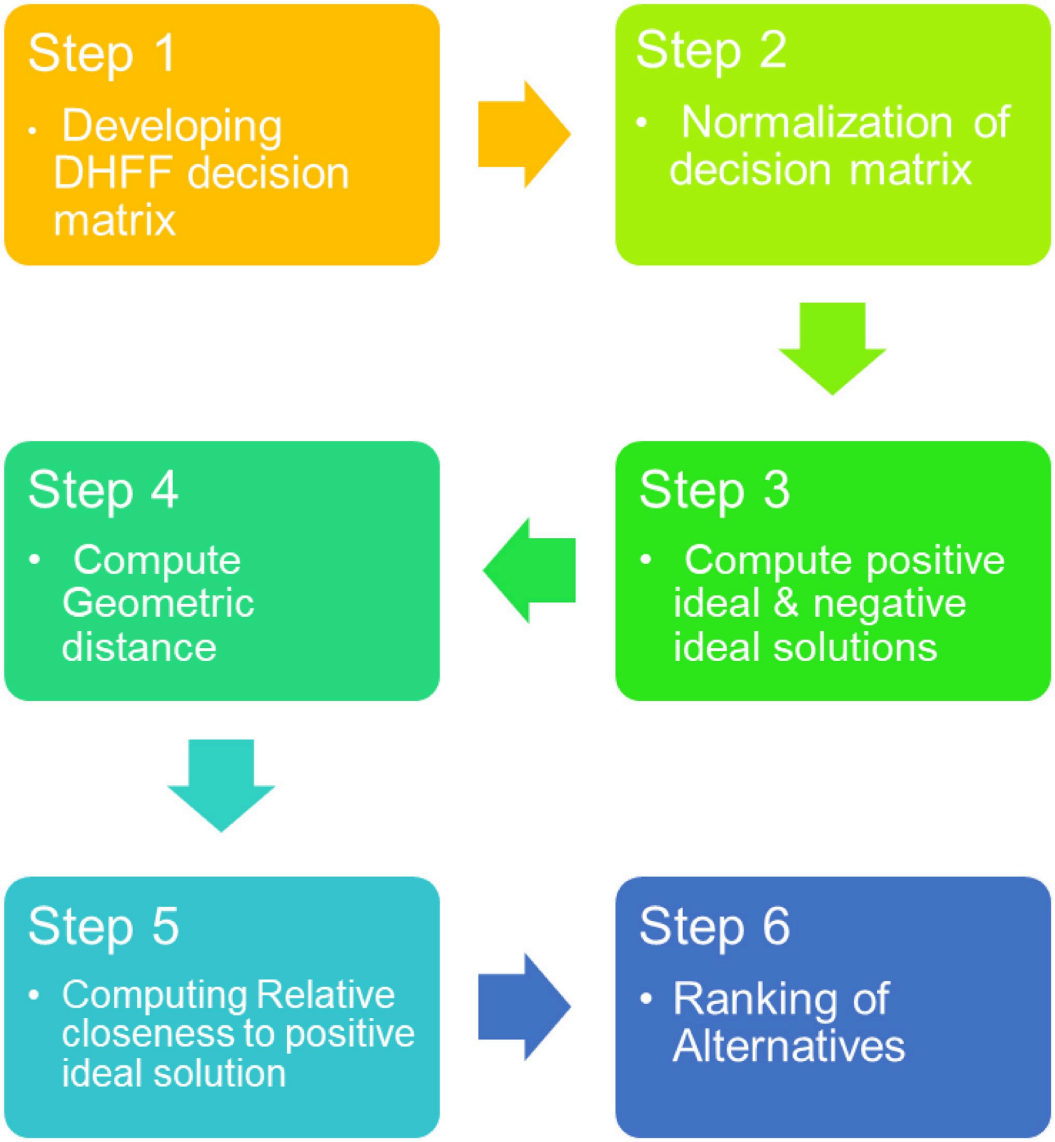
Flowchart of TOPSIS for MCDM with DHFFS.

A decision making process of TOPSIS is developed in dual-hesitant fermatean fuzzy set environment to solve the MCDM problems. A detailed step-wise flowchart of this model is presented in Fig 2. The steps of the decision-making process are given:

Step 1: Develop a dual-hesitant fermatean fuzzy decision matrix in which the alternatives are estimated relative to criteria set.
Step 2: Wang [61] describe the normalization of decision matrix that concept is used here.
Step 3: Relative to available decision matrix normalize the reference points *Δ*^+^, *Δ*^−^, for positive ideal (PI) solution $\Delta^{+}=\left(\sigma_{1}^{+},\sigma_{2}^{+},\sigma_{3}^{+},\ldots ,\sigma_{n}^{+}\right)$ and for negative ideal (NI) solution $\Delta^{-}=\left(\tau_{1}^{-},\tau_{2}^{-},\tau_{3}^{-},\ldots ,\tau_{n}^{-}\right)$ as discussed in [62].
Step 4: Geometric distance is measured in this step. Geometric distance between each alternative *Δ*_*i*_ and *Δ*^+^ is as:
  $$\begin{array}{c} \hat{D}\left(\Delta_{i},\Delta^{+}\right)=\sum_{\hat{j}=1}^{n}\omega_{i}\hat{D}\left(\left(\hbar_{i\hat{j}},{\hat{g}}_{i\hat{j}}\right),\Delta^{+}\right)=\sum_{\hat{j}=1}^{n}\omega_{i}\hat{D}\left(\left(\hbar_{i\hat{j}},{\hat{g}}_{i\hat{j}}\right),\left(\left\{1\right\},\left\{0\right\}\right)\right) \end{array}$$ (0.26)
  $$\begin{array}{c} =\sum_{\hat{j}=1}^{n}\omega_{i}{\left(\frac{1}{3}(\frac{1}{\#\hbar_{i\hat{j}}}\sum_{\sigma \in \hbar_{i\hat{j}}}{\left(1-\sigma^{3}\right)}^{\beta }+\frac{1}{\#{\hat{g}}_{i\hat{j}}}\sum_{\tau \in {\hat{g}}_{i\hat{j}}}\tau^{3\beta }+{\left(1-\left(\frac{1}{\#\hbar_{i\hat{j}}}\sum_{\sigma \in \hbar_{i\hat{j}}}\sigma^{3}+\frac{1}{\#{\hat{g}}_{i\hat{j}}}\sum_{\tau \in {\hat{g}}_{i\hat{j}}}\tau^{3}\right)\right)}^{\beta })\right)}^{\frac{1}{\beta }} \end{array}$$ (0.27)
  Geometric distance between each alternative *Δ*_*i*_ and *Δ*^−^ is as:
  $$\begin{array}{c} \hat{D}\left(\Delta_{i},\Delta^{-}\right)=\sum_{\hat{j}=1}^{n}\omega_{i}\hat{D}\left(\left(\hbar_{i\hat{j}},{\hat{g}}_{i\hat{j}}\right),\Delta^{-}\right)=\sum_{\hat{j}=1}^{n}\omega_{i}\hat{D}\left(\left(\hbar_{i\hat{j}},{\hat{g}}_{i\hat{j}}\right),\left(\left\{0\right\},\left\{1\right\}\right)\right) \end{array}$$ (0.28)
  $$\begin{array}{c} =\sum_{\hat{j}=1}^{n}\omega_{i}{\left(\frac{1}{3}(\frac{1}{\#\hbar_{i\hat{j}}}\sum_{\sigma \in \hbar_{i\hat{j}}}\sigma^{3\beta }+\frac{1}{\#{\hat{g}}_{i\hat{j}}}\sum_{\tau \in {\hat{g}}_{i\hat{j}}}{\left(1-\tau^{3}\right)}^{\beta }+{\left(1-\left(\frac{1}{\#\hbar_{i\hat{j}}}\sum_{\sigma \in \hbar_{i\hat{j}}}\sigma^{3}+\frac{1}{\#{\hat{g}}_{i\hat{j}}}\sum_{\tau \in {\hat{g}}_{i\hat{j}}}\tau^{3}\right)\right)}^{\beta })\right)}^{\frac{1}{\beta }} \end{array}$$ (0.29)
Step 5: Computing of relative closeness (*C*_*r*_) of *Δ*_*i*_ according to the PI solution *Δ*^+^ as,
  $$\begin{array}{c} C_{r}\left(\Delta_{i}\right)=\frac{\hat{D}\left(\Delta_{i},\Delta^{-}\right)}{\hat{D}\left(\Delta_{i},\Delta^{-}\right)+\hat{D}\left(\Delta_{i},\Delta^{+}\right)} \end{array}$$ (0.30)
Step 6: Lastly, ranking is done according to *C*_*r*_(*Δ*_*i*_), (*i* = 1, 2, ⋯, *n*).

## Practical-case

Pakistan is one of the countries with the highest natural gas reserves. The biggest natural gas field, making up 6% of the total, is in Balochistan’s Sui region. The country has proven reserves that are 12 times its yearly use. Therefore, it has around 12 years’ worth of gas remaining. Since there has been a serious natural gas scarcity in Pakistan for the past several years, the country has begun to rely heavily on liquefied natural gas (LNG). The government must investigate other energy sources to preserve the environment and cut costs associated with LNG imports. So, to overcome this energy crisis (natural gas shortage), the government must have a proper solution. To provide an uninterrupted gas supply to domestic consumers and industries, Pakistan’s energy sector needs to make an agreement with any country for a gas pipeline. So, to overcome the country’s gas crisis in the coming years. For that, there is a list of a few countries with which Pakistan can agree to gas pipelines. Let *Δ* = {*Δ*_1_, *Δ*_2_, *Δ*_3_, *Δ*_4_, *Δ*_5_} be a set of alternatives and *Δ*_1_ = Iran, *Δ*_2_ = Turkmenistan, *Δ*_3_ = Qatar, *Δ*_4_ = Russia, *Δ*_5_ = Saudi Arabia and here are few criteria which are to be considered while agreeing ∁_1_ = quality and quantity of resource, ∁_2_ = trade and taxation systems, ∁_3_ = infrastructure and security, ∁_4_ = economical, criteria set is ∁ = {∁_1_, ∁_2_, ∁_3_, ∁_4_}. Evaluation values to these above sets are demonstrated in Table 3 based on the DHFFS environment. The weight vector of given criteria is ⋓ = {.1, .35, .25, .3 }^*T*^.

**Table 3:** Dual-hesitant fermatean fuzzy decision matrix.

	∁_1_	∁_2_	∁_3_	∁_4_
*Δ*_1_	({.6,.8}, {.7})	({.3,.4}, {.5,.6})	({.4,.5}, {.8})	({.7}, {.5})
*Δ*_2_	({.6}, {.5,.8})	({.4,.5}, {.6})	({.2,.6}, {.6})	({.6,.7}, {.7})
*Δ*_3_	({.7,.8}, {.5})	({.5}, {.6,.9})	({.6}, {.3,.4})	({.5,.9}, {.5})
*Δ*_4_	({.8}, {.4})	({.8,.9}, {.2})	({.5,.3}, {.5})	({.4}, {.3,.4})
*Δ*_5_	({.1,.3}, {.9})	({.3}, {.8})	({.4}, {.7})	({.6,.8}, {.7,.6})

In the following, the most desirable country for gas pipeline agreement selection, we utilize the DHFFHWA and DHFFHWG operators to evaluate the MCDM problem by DHFFNs based on the following procedure.

Step 1. Overall preference values *♇*_*i*_ are acquired of the countries *Δ*_*i*_, (*i* = 1, 2, 3, 4, 5) based on information provided in decision matrix Table 3. Take *β* = 3, and solve for alternative 1, we have
  $$\begin{array}{c} =DHFFHWA\{\{(.6,.8),(.7)\},\{(.3,.4),(.5,.6)\},\{(.4,.5),(.8)\},\{(.7),(.5)\}\}\\ =\{.5281,.5463,.5441,.5613,.5622,.5784,.5764,.5919\},\{.5894,.6265\} \end{array}$$
Step 2. Calculating the $S_{⊺}\left({♇}_{i}\right)$ of overall preference values of dual-hesitant fermatean fuzzy preference values.
  $$\begin{array}{cc} S_{⊺}\left(\Delta_{1}\right) & =.6554,\;S_{⊺}\left(\Delta_{2}\right)=.4796,\;S_{⊺}\left(\Delta_{3}\right)=.6454,\\ & S_{⊺}\left(\Delta_{4}\right)=.7679,\;S_{⊺}\left(\Delta_{5}\right)=.4518. \end{array}$$
Step 3. Rank all the countries to get the best country for the future gas pipeline project i.e, *Δ*_4_ > *Δ*_1_ > *Δ*_3_ > *Δ*_2_ > *Δ*_5_. So, Russia is the best country to do agreement for the gas pipeline project.

We may create a method for solving multiple attribute decision-making issues using dual-hesitant Fermatean fuzzy information on the basis of the DHFFHWG operator, which can be summarised as follows:

Step 1. Overall preference values of countries *Δ*_*i*_, (*i* = 1, 2, 3, 4, 5) based on information provided in decision matrix. Take *β* = 3, and when solve for alternative 1, we have
  $$\begin{array}{cc} & =DHFFHWG\{\{(.6,.8),(.7)\},\{(.3,.4),(.5,.6)\},\{(.4,.5),(.8)\},\{(.7),(.5)\}\}\\ & =\{.6263,.6526\},\{.4525,.4783,.4982,.5259,.4687,.4952,.5156,.5439\} \end{array}$$
Step 2. Calculating the $S_{⊺}\left({♇}_{i}\right)$ of overall preference values of dual-hesitant fermatean fuzzy preference values.
  $$\begin{array}{cc} S_{⊺}\left(\Delta_{1}\right) & =.5254,\;S_{⊺}\left(\Delta_{2}\right)=.3661,\;S_{⊺}\left(\Delta_{3}\right)=.4873,\\ & S_{⊺}\left(\Delta_{4}\right)=.5585,\;S_{⊺}\left(\Delta_{5}\right)=.3422. \end{array}$$
Step 3. Rank all the countries to get the best country for the future gas pipeline project i.e, *Δ*_4_ > *Δ*_1_ > *Δ*_3_ > *Δ*_2_ > *Δ*_5_. So, Russia is the best country to do agreement for the gas pipeline project.

It is clear from the research above that while the total rating values of the alternatives are different, the ranking orders of the alternatives doesnot change when utilising two operators, respectively. *Δ*_4_ = *Russia* is the preferred country for agreement.

### Solving the numerical example with TOPSIS

The above mentioned numerical example can now be computed by using TOPSIS to show the validity and effectiveness of DHFFHWA and DHFFHWG. Based on the procedure given in section above evaluation of the given countries is carried on according to the available criteria, to get the best country for the supply of gas. Let the *β* = 3 then the procedure is as follows:

Step 1. A Dual-hesitant fermatean fuzzy decision matrix is shown in Table 3.
Step 2. Normalized decision matrix is given in Table 4.
Step 3. To obtain the normalization of reference points *Δ*^+^ and *Δ*^−^ based on Table 4.
  $$\begin{array}{cc} \Delta^{+} & =(\sigma_{1}^{+},\sigma_{2}^{+},\sigma_{3}^{+},\ldots ,\sigma_{n}^{+})\\ & =((\{1,1\},\{0,0\}),(\{1,1\},\{0,0\}),(\{1,1\},\{0,0\}),(\{1,1\},\{0,0\})),\\ \Delta^{-} & =(\tau_{1}^{-},\tau_{2}^{-},\tau_{3}^{-},\ldots ,\tau_{n}^{-})\\ & =((\{0,0\},\{1,1\}),(\{0,0\},\{1,1\}),(\{0,0\},\{1,1\}),(\{0,0\},\{1,1\})). \end{array}$$
Step 4. Geometric distance $\hat{D}\left(\Delta_{i},\Delta^{+}\right)$ and $\hat{D}\left(\Delta_{i},\Delta^{-}\right)$ is calculated in this step using Eqs [0.26] and [0.28] for the alternatives *Δ*_*i*_ = {1, 2, ⋯, 5} and the results are shown in Table 5.
Step 5. Compute the relative closeness of alternative *Δ*_*i*_ according to the PI solution *Δ*^+^.
Step 6. Lastly, rank is determined of all alternatives (countries) is done *Δ*_4_ > *Δ*_3_ > *Δ*_1_ > *Δ*_2_ > *Δ*_5_ obtain from Table 6, to choose the best option, arrange the relative closeness values you obtained in the previous step.i.e, *Δ*_4_.

**Table 4:** Dual-hesitant fermatean fuzzy normalized decision matrix.

	∁_1_	∁_2_	∁_3_	∁_4_
*Δ*_1_	({.6,.8}, {.7,.7})	({.3,.4}, {.5,.6})	({.4,.5}, {.8,.8})	({.7,.7}, {.5,.5})
*Δ*_2_	({.6,.6}, {.5,.8})	({.4,.5}, {.6,.6})	({.2,.6}, {.6,.6})	({.6,.7}, {.7,.7})
*Δ*_3_	({.7,.8}, {.5,.5})	({.5,.5}, {.6,.9})	({.6,.6}, {.3,.4})	({.5,.9}, {.5,.5})
*Δ*_4_	({.8,.8}, {.4,.4})	({.8,.9}, {.2,.2})	({.5,.3}, {.5,.5})	({.4,.4}, {.3,.4})
*Δ*_5_	({.1,.3}, {.9,.9})	({.3,.3}, {.8,.8})	({.4,.4}, {.7,.7})	({.6,.8}, {.7,.6})

**Table 5:** Geometric distances of alternatives.

Geometric distance	*Δ*_1_	*Δ*_2_	*Δ*_3_	*Δ*_4_	*Δ*_5_
$\hat{D}\left(\Delta ,\Delta^{+}\right)$	.7197	.7316	.6863	.6438	.7439
$\hat{D}\left(\Delta ,\Delta^{-}\right)$	.5929	.5728	.6244	.7594	.4703

**Table 6:** Closeness co-efficients of alternatives.

*Δ*_*i*_	*Δ*_1_	*Δ*_2_	*Δ*_3_	*Δ*_4_	*Δ*_5_
*C*_*r*_(*Δ*_*i*_)	.4517	.4391	.4763	.5411	.3873

## Comparative analysis

**Fig 3:**
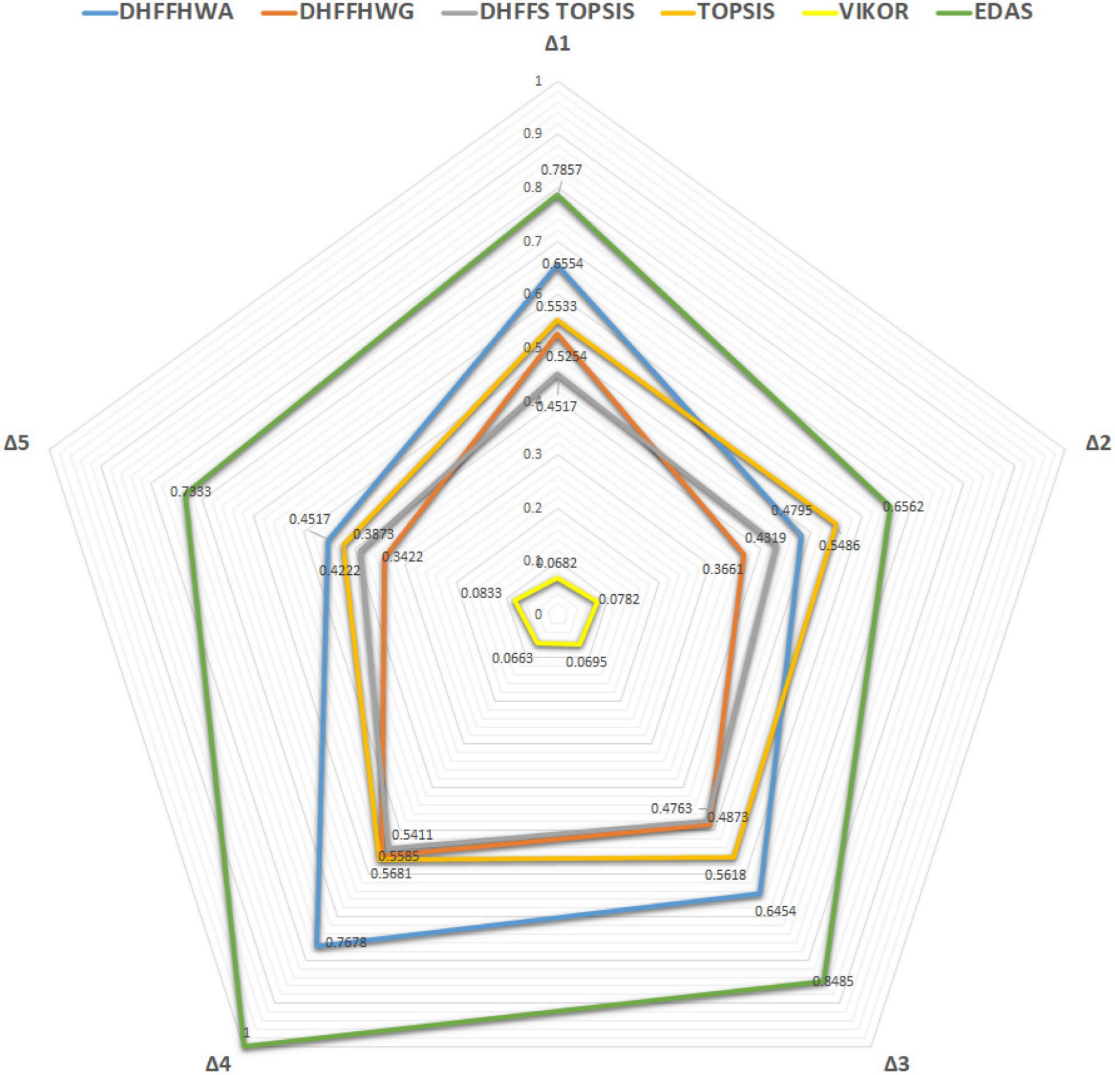
Ranking comparison of proposed operators and DHFFS-TOPSIS with traditional MCDM methods.

Fig 3 displays the various ranks of the countries to be considered for agreement in light of the comparison just given in Table 7. When comparing the rankings of the given alternatives applying the above methods, it is possible to state that the results do not differ essentially, mainly since the first choice is determined most compulsively. In every case, the DHFFS methods select *Δ*_4_ as the best option and agree closely with each other in the rankings of the other choices, not only for DHFFHWA and DHFFHWG but also when compared to the other MCDM methods explored in this paper, such as TOPSIS, VIKOR, and EDAS.

**Table 7:** Ranking Comparison of DHFFHWOs based on proposed operators and DHFFS-TOPSIS.

Methods	*Δ*_1_	*Δ*_2_	*Δ*_3_	*Δ*_4_	*Δ*_5_	Ranking orders
DHFFHWA	.6554	.4796	.6454	.7679	.4518	*Δ*_4_ > *Δ*_1_ > *Δ*_3_ > *Δ*_2_ > *Δ*_5_
DHFFHWG	.5254	.3661	.4873	.5585	.3422	*Δ*_4_ > *Δ*_1_ > *Δ*_3_ > *Δ*_2_ > *Δ*_5_
DHFFS-TOPSIS	.4517	.4391	.4763	.5411	.3873	*Δ*_4_ > *Δ*_3_ > *Δ*_1_ > *Δ*_2_ > *Δ*_5_
TOPSIS [63]	.5533	.5486	.5618	.5681	.4222	*Δ*_4_ > *Δ*_3_ > *Δ*_1_ > *Δ*_2_ > *Δ*_5_
VIKOR [64]	.0682	.0782	.0695	.0663	.0833	*Δ*_4_ > *Δ*_1_ > *Δ*_3_ > *Δ*_2_ > *Δ*_5_
EDAS [65]	.7857	.6562	.8485	1.000	.7333	*Δ*_4_ > *Δ*_3_ > *Δ*_1_ > *Δ*_5_ > *Δ*_2_

Although the DHFFS-TOPSIS in the ranking of *Δ*_1_ and *Δ*_3_ is slightly different from DHFFHWA and DHFFHWG, but it is similar to the traditional TOPSIS, which proves the correctness of expanding traditional methods into the dual-hesitant fermatean fuzzy environment. VIKOR which also prioritizes compromise solutions, places *Δ*_4_ in the top position, and the VIKOR method assigns numbers to the values of *Q*_*i*_, where the lowest *Q*_*i*_ rating corresponds to the highest-ranking option. However, it maintains a specific ranking hierarchy where *Δ*_4_ is chosen as the best suited as established by other methods but with slight shifts such that *Δ*_1_ outranked *Δ*_3_. This indicates VIKOR’s ability to respond to the variance in the benchmark between the best and worst options, though overall concurring with other ranking methods that *Δ*_4_ is superior.

EDAS also identifies *Δ*_4_ as having the highest preference, like the method introduced before; nevertheless, its scoring categorizes *Δ*_3_ superior to *Δ*_1_ and *Δ*_2_ due to its unique assessment system. Despite the slight variations in the ranks, it can be seen that there is a consensus on the ranking of the option (*Δ*_4_) in all the approaches, which tends to support the credibility of the DHFFS framework for handling decision-making problems. This similarity across the different methods not only supports the urging for the utilization of the DHFFS in complex decision-making but also shows that the adjustment of the common MCDM approaches to the context of the DHFFS does not necessarily weaken the decision-making consistency.

## Sensitivity analysis

This section focuses on the sensitivity analysis of criteria weights and parameter values to the proposed DHFFS-TOPSIS method. The forecast also shows how changes in criteria weights and parameter *β* impact the ranking of the alternatives, and it illustrates that the method is very flexible.

**(i) Baseline analysis:** The DHFFS-TOPSIS method was initially applied with the default criteria weights, resulting in the rankings in Table 8. The best alternative is *Δ*_4_, followed by *Δ*_3_, *Δ*_1_, *Δ*_2_, and *Δ*_5_. This baseline serves as the reference point for subsequent sensitivity tests.
**(ii) Equal weights of criteria:** To assess the influence of equal weighting, all the criteria weights were pre-scaled by the factor of 0.25. The findings show that *Δ*_4_ has stood out as the most preferred alternative, thus pointing to an unchanged order among the alternatives. This also implies that the proposed DHFFS-TOPSIS method is not much affected by the equal importance of criteria as the optimum results are obtained.
**(iii) Minor changes in criteria weight:** For this purpose, minor adjustments to the criteria weights were made to evaluate the shift in the ranking. The analysis of results also shows that the ranking order is unaffected, and *Δ*_4_ continues to come at the top. This implies that using the DHFFS-TOPSIS method, results are consistent and robust to variations in weight assigned to each criterion, promoting consistency irrespective of fluctuations in the weights assigned to criteria.
**(iv) Increased weight for criterion ⋓_3_:** Subsequently, the weight of criterion ⋓_3_ was adjusted to 0.7, and it is necessary to note the ranked outputs. The study’s findings also indicate that out of the five alternatives, *Δ*_3_ rose to the top and replaced first-place *Δ*_4_. This outcome supports the fact that the method is inclined towards the weight of ⋓_3_ and can change faster depending on the weight of individual criteria.
**(v) Variation in parameter *β*:** Lastly, to test the effect of the mentioned parameter, the value of *β* was changed, and analysis was done. For the list, when *β* was set to 8, the novice subjects were ranked similarly to the baseline, just swapping *Δ*_2_ and *Δ*_1_ in the rankings. This implies that, although the DHFFS-TOPSIS method is stable irrespective of any change in *β*, varying some of the parameters can lead to a slight change in the ranking of the alternatives.

**Table 8:** Sensitivity analysis of DHFFS-TOPSIS.

No.	Substituted values	Results	Rankings
1	DHFFS-TOPSIS ⋓ = {.10, .35, .25, .30}^*T*^	0.4517	*Δ*_4_ > *Δ*_3_ > *Δ*_1_ > *Δ*_2_ > *Δ*_5_
		0.4319	
		0.4763	
		0.5411	
		0.3873	
2	Same weights of criteria ⋓ = {.25,.25,.25, .25}^*T*^	0.4486	*Δ*_4_ > *Δ*_3_ > *Δ*_1_ > *Δ*_2_ > *Δ*_5_
		0.4410	
		0.4935	
		0.5441	
		0.3642	
3	Minor changes in weights ⋓ = {.10, .30, .20, .30}^*T*^	0.4571	*Δ*_4_ > *Δ*_1_ > *Δ*_2_ > *Δ*_3_ > *Δ*_5_
		0.4381	
		0.4789	
		0.5401	
		0.3887	
4	Increased weight for criterion ⋓_3_ ⋓ = {.10, .10, .70, .10}^*T*^	0.3841	*Δ*_3_ > *Δ*_4_ > *Δ*_2_ > *Δ*_5_ > *Δ*_1_
		0.4436	
		0.5045	
		0.4991	
		0.3975	
5	Same ⋓ and *β* = 8 ⋓ = {.25, .25, .25, .25}^T^	0.4566	*Δ*_4_ > *Δ*_3_ > *Δ*_2_ > *Δ*_1_ > *Δ*_5_
		0.4574	
		0.5091	
		0.5597	
		0.3677	

**Fig 4:**
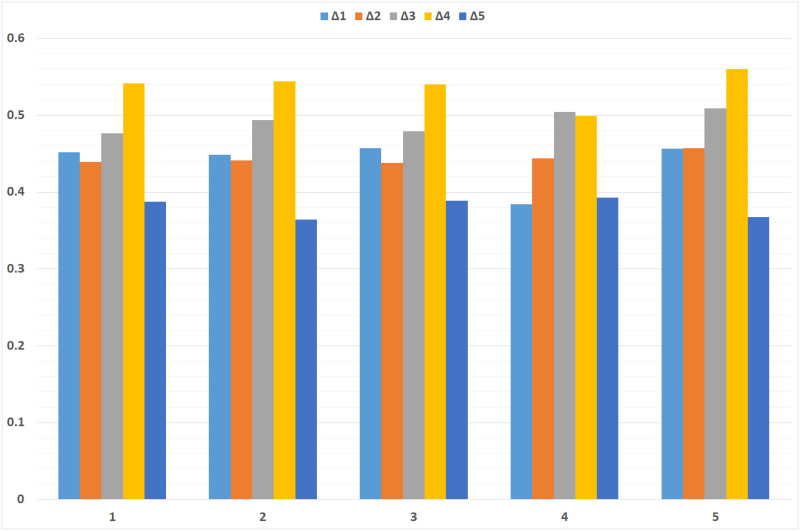
Sensitivity analysis of DHFFS-TOPSIS.

The findings of the sensitivity analysis summarized in Table 8 show that the DHFFS-TOPSIS method is highly stable and manageable. It reacts accordingly to variations in criteria weights and parameters. Hence, people can obtain precise outcomes in distinctive circumstances. The graphical comparison of sensitivity analysis is shown in Fig 4.

### Rank reversal technique

Rank reversal is a process in which the implementation of a specific approach of MCDM leads to a change in some of the alternatives’ positions in the ranking scale as a result of the insertion or removal of another one. This is a crucial aspect of the decision-making process, which can affect reliability and standardization. Hence, it provides a basis for assessing MCDM methods. In an ideal world, a good MCDM technique should not include rank reversal, which ensures that rank assignments are immune to change by adding more choices. Thus, rank reversal has to be considered as one of the focal issues that need to be addressed with the purpose of enhancing the credibility and potentially the positive acceptance of any MCDM system. Rank reversal is a phenomenon that arises when applying some of the methods that lead to the mistaken ordering of alternatives based on different criteria, and that is why such methods can lead to reasonably problematic decision-making. Preventing rank reversal in a method guarantees the methods’ reliability and, therefore, increases its reliability to the decision-makers.

To determine our suggested method’s resistance to rank reversal, we added a new alternative, *Δ*_6_, to the existing set of alternatives (*Δ*_1_, *Δ*_2_, *Δ*_3_, *Δ*_4_, and *Δ*_5_), as shown in Table 9. The purpose was to see if the addition of *Δ*_6_ would affect the ranks of the current alternatives. Initially, the options were sorted as follows: *Δ*_4_ > *Δ*_3_ > *Δ*_1_ > *Δ*_2_ > *Δ*_5_. These rankings were obtained using our method based on the original set of criteria and provided weight vector. We added a new option, *Δ*_6_, and re-evaluated the ranks with DHFFS-TOPSIS. The performance values for *Δ*_6_ were standardized and integrated into the current dataset with new ranking order as *Δ*_4_ > *Δ*_3_ > *Δ*_6_ > *Δ*_1_ > *Δ*_2_ > *Δ*_5_. The re-evaluation process entailed recalculating the distances and using the same methodology to establish the new rankings.

**Table 9:** Dual-hesitant fermatean fuzzy normalized decision matrix with *Δ*_6_.

	∁_1_	∁_2_	∁_3_	∁_4_
*Δ*_1_	({.6,.8}, {.7,.7})	({.3,.4}, {.5,.6})	({.4,.5}, {.8,.8})	({.7,.7}, {.5,.5})
*Δ*_2_	({.6,.6}, {.5,.8})	({.4,.5}, {.6,.6})	({.2,.6}, {.6,.6})	({.6,.7}, {.7,.7})
*Δ*_3_	({.7,.8}, {.5,.5})	({.5,.5}, {.6,.9})	({.6,.6}, {.3,.4})	({.5,.9}, {.5,.5})
*Δ*_4_	({.8,.8}, {.4,.4})	({.8,.9}, {.2,.2})	({.5,.3}, {.5,.5})	({.4,.4}, {.3,.4})
*Δ*_5_	({.1,.3}, {.9,.9})	({.3,.3}, {.8,.8})	({.4,.4}, {.7,.7})	({.6,.8}, {.7,.6})
*Δ*_6_	({.4,.3}, {.5,.8})	({.5,.4}, {.4,.6})	({.5,.4}, {.4,.5})	({.5,.6}, {.4,.7})

After introducing *Δ*_6_, the ranks of the original alternatives (*Δ*_1_, *Δ*_2_, *Δ*_3_, *Δ*_4_, and *Δ*_5_) remained unaltered. The new alternative, *Δ*_6_, was ranked third. The final rankings were *Δ*_4_ > *Δ*_3_ > *Δ*_6_ > *Δ*_1_ > *Δ*_2_ > *Δ*_5_. Despite the insertion of *Δ*_6_, the original ranks remained stable, indicating that our technique does not suffer from rank reversal. This highlights resilience and reliability of our proposed approach, guaranteeing that the decision-making process remains consistent even when the collection of possibilities evolves.

## Scalability discussion

The case study related to the country selection for the gas pipeline project revealed and proved the effectiveness of the proposed DHFFSs model and its aggregation operators, such as DHFFHWA and DHFFHWG. Nevertheless, it can go much further than this application and be used in other spheres and on a larger scale. It is generic and can be applied to various MCDM issues. For example, it can be applied in the supply chain where the suppliers can be graded on quality, reliability, cost, and the environment. It can be applicable in healthcare to compare the efficiency of treatments or healthcare providers in terms of benefit, harm, cost, and patient satisfaction. In addition, while analyzing the results, it can be used in urban planning, for example, choosing the location for new facilities or comparative analysis of transport solutions according to the costs, impact on the environment, and the people’s acceptance. This aspect is beneficial in the abovementioned situations where accurate data might be unavailable since DHFFSs can easily accommodate indicators of uncertainty and hesitation.

Concerning the applicability to larger scales, it is possible to comprehend that the DHFFS approach can comparatively consider a broader range of alternatives and criteria. The methodology can be extended to situations with many more alternatives, with the major impact being the number of comparisons and computations done. This challenge can be addressed using proper computations as technology plays a central role in this challenge. Also, the model can include more criteria, enabling the assessment of contenders along more dimensions of the decision problem. The mathematical basis of DHFFSs allows the processing of high-dimensional data, which makes it possible to use the model to represent the high-level relations and hesitancies within the opponent’s big data.

The presence of many alternatives and criteria increases the number of calculations needed. These issues can be addressed by optimizing the algorithms and software techniques involved so that the model is still practical to deploy at scale. Therefore, a proposed DHFFS based MCDM model indicates the advantages in addressing uncertainty and hesitancy, making the model suitable to solve diverse problems related to industries and scales. Also, supplementing the application of DHFFS with other decision-making tools, such as TOPSIS, increases its effectiveness and reliability. It makes the integration possible to cross check information and enhance the decision-making matrix to filter the right results.

## Managerial implications

The results of this research provide an outstanding practical value for the managers and decision-makers in Pakistan’s energy sector, especially in terms of international partners for the gas pipeline agreements. The described DHFFS-TOPSIS method gives sound ground for analyzing and ranking potential countries according to different criteria that play an essential role in decision-making concerning such types of agreements. The method above of DHFFS-TOPSIS makes it possible for managers to examine issues relating to the quality and quantity of resources objectively, as well as trade, taxation systems, infrastructure, security, and economic factors linked to each partner country. Such an extensive appraisal helps to identify whether the chosen country complies with Pakistan’s long-term energy vision and sustainability factors. This is because the approach involved in the method eases uncertainties and hesitations that are likely to be encountered in the decision-making process and are not considered by other conventional methods, such as the TOPSIS method. This approach minimizes the probability of making wrong decisions driven by limited or vague information, which prides itself on a clumsy and unstable industry such as energy. Managers can benefit from our method’s rank reversal resistance, which provides stable and trustworthy rankings, leading to more confident and reliable decisions. This way, the managers can rank the countries based on their economic and infrastructural compatibility with Pakistan’s requirements and use the negotiation power to get better terms and optimal costs for the gas pipeline project. This is especially vital, bearing in mind that the country has been importing LNG, and there is a need to look for cheap energy sources. The findings derived from the analysis reveal that Russia is the most appropriate candidate for a gas pipeline accord. It proves beneficial for the managers to direct all the diplomatic and negotiation steps towards forming a strategic partnership with Russia that can further induce better terms and a secure energy supply to Pakistan.

Comparing hesitation and membership degrees, the DHFFS-TOPSIS method can help managers better understand the alternatives evaluation. The simultaneous consideration of both types of information improves the decision-making and accuracy and reliability of the process, mainly in conditions of uncertainty or paradoxical data. The DHFFS-TOPSIS method can be applied across different business fields, including finance, the health sector, manufacturing, logistics, and the like. This method can provide managers in these industries a way to solve decision-making problems, which have many criteria, including selecting a supplier, evaluating a project, or assessing a risk where the ordinary approach may not be effective. Because the DHFFS-TOPSIS method used here concerns the revelation of the decision-making process, it improves communication between various stakeholders. Because of this, managers can easily defend why they have made decisions, and other stakeholders will support this.

## Limitations and future work

Its limitation is that the study focuses on selected distance measures and aggregation operators only, which may influence the availability of the outcomes. Thus, the proved hypothesis deals with a definite set of countries and criteria and, therefore, it can be less relevant to other countries. These limitations include the fact that although the study identifies specific Hamacher aggregation operators on which the proposed methods hinge, there might be limited flexibility in such methods when applied to several types of decision-making processes. Future work should expand the literature search towards as many different methodologies as possible, and the proposed methods must be tried on various data sets from different areas. Besides, since the proposed approach is based on DHFFSs, studies extending the family of fuzzy set theories may be investigated to enhance the proposed methods’ scope. Increasing coverage to other fuzzy set theories and integrating state-of-the-art computational tools like artificial intelligence and machine learning could also improve the decision-making process. Moreover, casting these methods across various disciplines, including medicine and health care, business and finance, and ecology and environmental issues, it is possible to find new approaches to solving multifaceted and sophisticated problems.

## Conclusion

This paper proposes the DHFFS along with the DHFFHWA and DHFFHWG operators. It connects fuzzy arithmetic with hesitation to apply the Fermatean fuzzy set to complex computational tasks more efficiently. To prove its effectiveness, the DHFFS framework was illustrated by a real-world case, choosing a country to construct a gas pipeline. Based on the comparative analysis, it is evident that methods under the DHFFS, namely the DHFFHWA, DHFFHWG, and DHFFS-TOPSIS, have a significant correlation with the traditional MCDM techniques such as the TOPSIS, VIKOR, and EDAS techniques. They provide similarity in the ranks they produce, with slight changes in some instances. This is further evidence of the strong positive correlation of the DHFFS approach in giving dependable information to the decision-maker. Sensitivity analysis also proves that, the method introduced as DHFFS-TOPSIS is sensitive with the changes in criteria weights and parameter values, therefore approval for its validity and flexibility is justified. The method’s resistance to rank reversal further ensures that decision-making remains consistent and reliable, even in dynamic environments with evolving alternatives. The discussion of scalability stresses that this model, DHFFS, can solve even bigger and more complex problems in numerous fields. In general, the DHFFS framework can be appreciated as a progressive contribution to the fuzzy set theory, enhancing the management of dual hesitancy and uncertainty in the context of MCDM. The case study of the gas pipeline project is an example of the use of the proposed method. It proves its efficiency and applicability, which is why it can be considered an exhaustive method for effective decision-making by managers and decision-makers in organizations and companies dealing with uncertainty.
